# Real-world effectiveness of tofacitinib in monotherapy or in combination with csDMARDs in patients with rheumatoid arthritis: A collaboration between Dutch registries (TOFADUDAP study)

**DOI:** 10.1007/s10067-026-08362-x

**Published:** 2026-08-19

**Authors:** Isabell S. Nevins, Herman Kasper Glas, Floor Reimann, Reinhard Bos, Marieke P. D. de Buck, K. Wiepke Drossaers-Bakker, Mirjam C. Hegeman, Tom W. J. Huizinga, Sytske Anne Bergstra

**Affiliations:** 1 Department of Rheumatology, Leiden University Medical Center, Albinusdreef 2, 2333 ZB Leiden, the Netherlands; 2 Department of Rheumatology, Reumazorg Zuid West Nederland, Goes, the Netherlands; 3 Department of Rheumatology, Frisius Medisch Centrum, Leeuwarden, the Netherlands; 4 Department of Rheumatology, Haaglanden Medisch Centrum, The Hague, the Netherlands; 5 Department of Rheumatology, Medisch Spectrum Twente, Enschede, the Netherlands

**Keywords:** Antibody, Biological disease modifying anti-rheumatic drugs, Drug survival, Elderly, Real-world data, Rheumatoid Arthritis, Targeted synthetic disease modifying anti-rheumatic drugs

## Abstract

**Objective:**

To compare real-world effectiveness of tofacitinib monotherapy to adalimumab monotherapy, and tofacitinib and adalimumab in combination with csDMARDs in Rheumatoid Arthritis (RA) subgroups.

**Methods:**

Retrospective routine care data was used from six Dutch cohorts for RA patients who initiated tofacitinib or adalimumab between 2001 and 2022. Drug survival of tofacitinib monotherapy (*n* = 229) was compared with that of tofacitinib combination-therapy (*n* = 179), adalimumab monotherapy (*n* = 1019) and adalimumab combination-therapy (*n* = 1361) using Kaplan–Meier curves and Cox regression. Drug survival was evaluated as an indirect measure of treatment persistence. Exploratory analyses assessed effect modifications by anti-citrullinated protein antibody (ACPA) status and age at treatment onset in both models.

**Results:**

Patients who started tofacitinib monotherapy were older compared to other treatment groups (61 vs 55, 58 and 58 years) and had used at least one previous b/tsDMARD more often compared to the adalimumab treatment groups (36% vs 41%, 19% and 13%). Compared to tofacitinib monotherapy, the risk of drug discontinuation was similar for tofacitinib combination-therapy (aHR 1.22, 95% CI: 0.96–1.54) and adalimumab monotherapy (aHR 0.89, 95% CI: 0.73–1.09) but lower for adalimumab combination-therapy (aHR 0.78, 95% CI: 0.63–0.96). Older age was associated with significantly higher risk of drug discontinuation. No effect modification by ACPA was observed.

**Conclusion:**

Based on real-world data from six Dutch cohorts, drug discontinuation was higher for tofacitinib than for adalimumab, with similar outcomes with or without csDMARDs. Older age was associated with a higher risk of drug discontinuation independently of treatment group, although this finding may be influenced by factors beyond treatment effectiveness.

**Table:** 

**Key Points** • *In a real-world setting tofacitinib was observed to have similar drug survival in mono- and combination therapy* • *ACPA status did not influence drug survival* • *Older age was related to a higher risk of drug discontinuation independently of treatment*

**Supplementary Information:**

The online version contains supplementary material available at https://doi.org/10.1007/s10067-026-08362-x.

## Introduction

The treatment landscape for rheumatoid arthritis (RA) has evolved significantly over time, most recently with the introduction of Janus kinase (JAK) inhibitors complementing biologic disease-modifying antirheumatic drugs (bDMARDs). Current EULAR recommendations, in line with a treat-to-target strategy, recommend both biologic (b) and targeted synthetic (ts) DMARDs as an effective and safe treatment option for RA patients who do not adequately respond to conventional synthetic Disease Modifying Antirheumatic drugs (csDMARDs). However, the variety of treatment mechanisms and absence of predictive factors for treatment success in individual patients make achieving the desired treatment response a process of trial and error, even within a structured treat-to-target approach [1].

In clinical practice, Tumor Necrosis Factor inhibitors (TNFi) are often the preferred choice of treatment due to extensive prescriber experience, established efficacy, safety and the increased affordability offered by biosimilars. Meanwhile, JAK inhibitors, classified as tsDMARDs, have shown comparable efficacy to bDMARDs and an acceptable safety profile when csDMARD therapy has failed [2–4]. Additionally, several randomized controlled trials (RCTs) have indicated that JAK inhibitors may be more effective when used as monotherapy compared to bDMARDs [1, 2, 5–10]. However, it remains to be determined whether the long-term clinical efficacy and persistence of JAK inhibitors are influenced by co-treatment with a csDMARD, and how their effectiveness compares to TNFi combination therapy in routine clinical settings [11–19]. To bridge the gap between a well-controlled trial and daily clinical practice, where the efficacy of a drug may differ – a phenomenon known as the efficacy-effectiveness gap – real world data studies are essential [20]. These studies are particularly important in light of recent safety concerns associated with JAK inhibitors, as they may help determine whether long term benefits differ across certain RA subgroups, such as anti-citrullinated protein antibody (ACPA) negative and elderly patients.

This study aimed to evaluate the real-world comparative effectiveness and persistence of tofacitinib monotherapy to tofacitinib combination therapy with a csDMARD, adalimumab monotherapy and adalimumab combination therapy using data from RA patients receiving standard care. Additionally, this study sought to investigate whether the effectiveness of these treatment options is influenced by ACPA status or patient age. Adalimumab was selected as the TNFi comparator due to its frequent use in clinical practice.

## Methods

### Patient selection

Data were collected from six longitudinal Dutch cohorts involving RA patients treated in routine care. The cohorts originated from the Leiden University Medical Center (LUMC), Haaglanden Medisch Centrum (HMC), Sint Maartenskliniek (SMK), Medisch Centrum Leeuwarden (MCL), Medisch Spectrum Twente (MST) and Reumazorg Zuid West Nederland (RZWN). From medical health records, ranging between 2000 and 2022, each site identified adults (≥ 18 years) with a clinical RA diagnosis who initiated tofacitinib or adalimumab and had at least one follow-up visit. Data included disease characteristics, treatment history, and disease activity (DAS28). As follow-up interval varied, typically visits occurred every four months according to Dutch guidelines. Because data were pseudo-anonymized and collected retrospectively from routine practice without a prespecified protocol, a waiver from informed consent was obtained.

### Outcome measures

The primary outcome measure was drug survival, based on start-and stop dates of tofacitinib/adalimumab recorded in medical files. To avoid misclassifying treatment discontinuation due to remission as failure, a DAS28 ≥ 2.6 at discontinuation was required. Sensitivity analyses included all-cause discontinuation, irrespective of DAS28. If stop dates were missing, drug use was assumed to continue until the start of another b/tsDMARD or cohort extraction date. Temporary discontinuation (< 3 months) was considered continuous use. If the stop date was missing and more than six months separated the last recorded visit date and cohort extraction date, patients were censored at six months after the last recorded visit. Patients were grouped into monotherapy or combination therapy at treatment start. Combination therapy was defined as csDMARD use at the time of or within ≤ 3 months after tofacitinib/adalimumab initiation and if a csDMARD was both (1) continued for 3 months and (2) used during ≥ 50% of the treatment duration. Temporary csDMARD interruptions (< 3 months) were still considered continuous use. This range was chosen as a pragmatic threshold to reflect sustained exposure in real-world practice while accounting for temporary interruptions and flexible dosing patterns.

### Statistical analysis

Baseline characteristics were compared across treatment groups, with tofacitinib monotherapy as reference group, using *T* tests, Mann–Whitney *U* tests or X^2^ tests. Tofacitinib monotherapy was chosen as reference group because this study aimed to examine difference in effectiveness of the addition of a csDMARD and TNFi mono- and combination therapy to tofacitinib monotherapy. If patients switched between adalimumab or tofacitinib treatment during the course of follow up, they were accounted for in both baseline treatment group descriptive statistics. Additionally, baseline characteristics within these treatment groups were compared between elderly versus younger patients at treatment (tofacitinib/adalimumab) onset and ACPA positive versus ACPA negative patients. Age cutoffs were made at ≥ 65 years, arbitrarily defining elderly patients, and at < 55 years arbitrarily defining younger patients. ACPA status was based on local tests and center specific laboratory cut-off values for ACPA positivity. Kaplan–Meier and Cox regression models were used to estimate drug retention, using tofacitinib monotherapy as the reference group and patient-level clustered standard errors to account for within-patient correlation. If patients switched between adalimumab or tofacitinib treatment during the course of follow up, they were accounted separately for both drug retention rates. Adjusting for competing drug survival was not considered because both events were assumed to be unordered and take place independently of one another. Analyses were adjusted for the potential confounders age, sex, smoking behavior, baseline DAS28, ACPA status, center of treatment and previous b/tsDMARD use. Exploratory analyses assessed effect modification by age group and ACPA status using interaction terms.

Significant interactions (*p* < 0.10) led to stratified analyses. Results were reported as hazard ratios (HRs) with 95% confidence intervals.

### Missing data

Missing data were assumed to be missing at random (MAR). Multiple imputation by chained equations with predictive mean matching was performed. The imputation model included all variables used in the analyses, including predictor, outcome and baseline covariates (Supplementary material 5, online resource). Appropriate regression models were applied depending on variable type (linear regression for continuous variables and logistic/multinomial regression for categorical variables. A total of 20 imputed datasets, was used for missing baseline covariates.

### Sensitivity analysis

To assess the influence of within patient correlation, a sensitivity analysis limited to the first b/tsDMARD treatment duration (adalimumab/tofacitinib) was conducted. To account for a potential confounding effect of drug availability, a sensitivity analysis was performed that only included patients starting adalimumab after 1–1–2009 and after 1–1–2012, since from 2009 onwards several bDMARDs with different modes of action were available and from 2012 onwards tofacitinib became available. A third sensitivity analysis, only including patients without prior b/tsDMARD use, was performed to assess the effect of line of treatment on drug survival. These sensitivity analyses were performed after multiple imputation. Due to the amount of missingness in prior b/tsDMARD use for tofacitinib, assumptions were explored after multiple imputation. These included the following assumptions in case of missingness: (1) patients had been prescribed no previous b/tsDMARDs before initiating tofacitinib, (2) patients had been prescribed at least one previous b/tsDMARD before initiating tofacitinib and (3) patients had been prescribed at least two or more previous b/tsDMARDs before initiating tofacitinib. Given tofacitinib’s common use as third/fourth line therapy, effect estimates were expected to fall amid these extremes.

All analyses were performed in STATA/SE 16.1.

## Results

### Patient characteristics

In the six participating hospitals, 2684 patients were identified who started tofacitinib and/or adalimumab for the first time, between 1 Juli 2001 and 20 December 2022, while being > 18 years old, and had more than one follow-up visit. These patients were included in the primary analyses (Supplementary Fig. S1.1, online resource). 408/2684 patients initiated tofacitinib and 2380/2684 patients initiated adalimumab during follow-up, and 104/2684 patients had initiated both tofacitinib and adalimumab at some time during follow-up. Among the included patients who initiated tofacitinib, 229/408 (56%) received monotherapy and 179/2408 (44%) received combination therapy with at least one csDMARD. Among patients who initiated adalimumab, 1019/2380 (43%) received monotherapy and 1361/2380 (57%) received combination therapy (Table 1). The total number of included patients per hospital site are listed in the Supplementary Table S1.1 (online resource).

**Table 1:** Baseline characteristics per treatment group

	TOFA monotherapy	TOFA + csDMARD	*P* value	ADA monotherapy	*P* value	ADA + csDMARD	*P* value
N, % of total	229 (8)	179 (6)		1019 (37)		1361 (49)	
Female, %	71	75	0.407	69	0.529	67	0.263
*Missingness, %*	*0*	*0*		*0.1*		*0.2*	
Age at start tofa, mean (SD)	61 (13)	55 (14)	** < 0.001**	NA		NA	
*Missingness, %*	*0*	*0*					
Age at start ada, mean (SD)	NA	NA		58 (14)	** < 0.001**	58 (13)	** < 0.001**
*Missingness, %*				*0*		*0.4*	
Number of previous b/tsDMARDs before start of tofa/ada, %							
*0*	25	27	0.702	80	** < 0.001**	88	** < 0.001**
*1*	12	19		16		11	
> *2*	24	22		3		2	
*Missingness, %*	*39*	*31*		*1*		*0*	
DAS28 at start of tofa/ada, mean (SD)	3.4 (1.4)	3.5 (1.3)	0.435	3.6 (1.5)	0.138	3.7 (1.5)	0.096
*Missingness, %*	*66*	*56*		*49*		*49*	
ACPA positivity, %	56	50	0.254	44	0.270	51	**0.002**
*Missingness, %*	*12*	*28*		*35*		*31*	
RF positivity, %	33	38	**0.004**	47	**0.006**	54	** < 0.001**
*Missingness, %*	*47*	*53*		*36*		*31*	
Current/ever smoker, %	20	30	0.782	28	0.297	31	0.676
*Missingness, %*	*68*	*51*		*53*		*49*	

*Ada*, adalimumab; *ADA* mono, adalimumab monotherapy; 95% CI, 95% confidence interval; *b/tsDMARDs*, biologic/targeted synthetic disease-modifying antirheumatic drugs; *csDMARD*, conventional synthetic disease-modifying antirheumatic drug; *DAS28*, disease activity score assessed using 28 joints, in case of missing values a new DAS28 score was calculated based on the individual components or the DAS28 score within a window of 4 weeks before baseline was carried forward; *NA*, Not Available; *RF +*, Rheumatoid Factor positive; Smoker, patients who were smokers or who had ever smoked; *TOFA*, tofacitinib; tofa, tofacitinib.

^a^P values reflect comparisons with TOFA monotherapy as reference group

Overall, baseline characteristics were comparable between patients treated with tofacitinib monotherapy and combination therapy (Table 1). The percentage of patients who were ACPA-positive did not differ and, at baseline, mean disease activity score (DAS28) was similar between tofacitinib mono- and combination therapy. Patients treated with tofacitinib monotherapy were on average older than those treated with tofacitinib combination therapy or adalimumab mono- or combination therapy (61 vs 55, 58 and 58 years). Furthermore, treatment was more often initiated after a higher number of previous used b/tsDMARDs. Baseline characteristics stratified by ACPA status and age at treatment onset (elderly vs young) within the treatment groups are shown in Supplementary Tables S1.2 and S1.3 (online resource).

### Primary analyses assessing drug survival per treatment group

The median year (interquartile range (IQR)) of initiation of tofacitinib monotherapy, tofacitinib combination therapy, adalimumab mono- and combination therapy was 2019 (2019–2020), 2019 (2019–2020), 2017 (2014–2019) and 2018 (2014–2019), respectively. The last follow-up visit was recorded on the 23rd of December 2022. Patients in both adalimumab treatment groups had a considerably longer treatment duration (maximum treatment duration of 17 years) than patients in the tofacitinib treatment groups (maximum treatment duration of 4.3 years). To enable better comparison of drug survival between treatment groups, the survival time of both adalimumab treatment groups were right censored when exceeding the maximum treatment duration of the tofacitinib treatment groups.

Also, to ensure that drug discontinuation was not mistaken for discontinuation due to sustained remission, all results on drug survival analyses were obtained using tofacitinib/adalimumab stop dates, with the requirement of a DAS ≥ 2.6. Out of the 184 censored cases in the tofacitinib treatment groups, 34 were due to DAS < 2.6 at the time of tofacitinib cessation. Whereas 214/1154 censored cases in the adalimumab treatment groups were due to DAS < 2.6 at the time of adalimumab cessation. The median follow up duration, including all patients after adalimumab treatment groups were right censored, was 3.3 years (IQR: 1.5–4.3 years). The median follow-up duration for patients treated with tofacitinib mono- and combination therapy and adalimumab mono- and combination therapy was 1.9 and 2.2, 4.3 and 4.3 years, respectively (*p* < 0.001). Tofacitinib was discontinued in 128 (56%) and 96 (54%) patients with tofacitinib mono- and combination therapy, and adalimumab was discontinued in 563 (55%) and 663 (49%) patients with adalimumab mono- and combination therapy, respectively (*p* < 0.001).

**Fig. 1:**
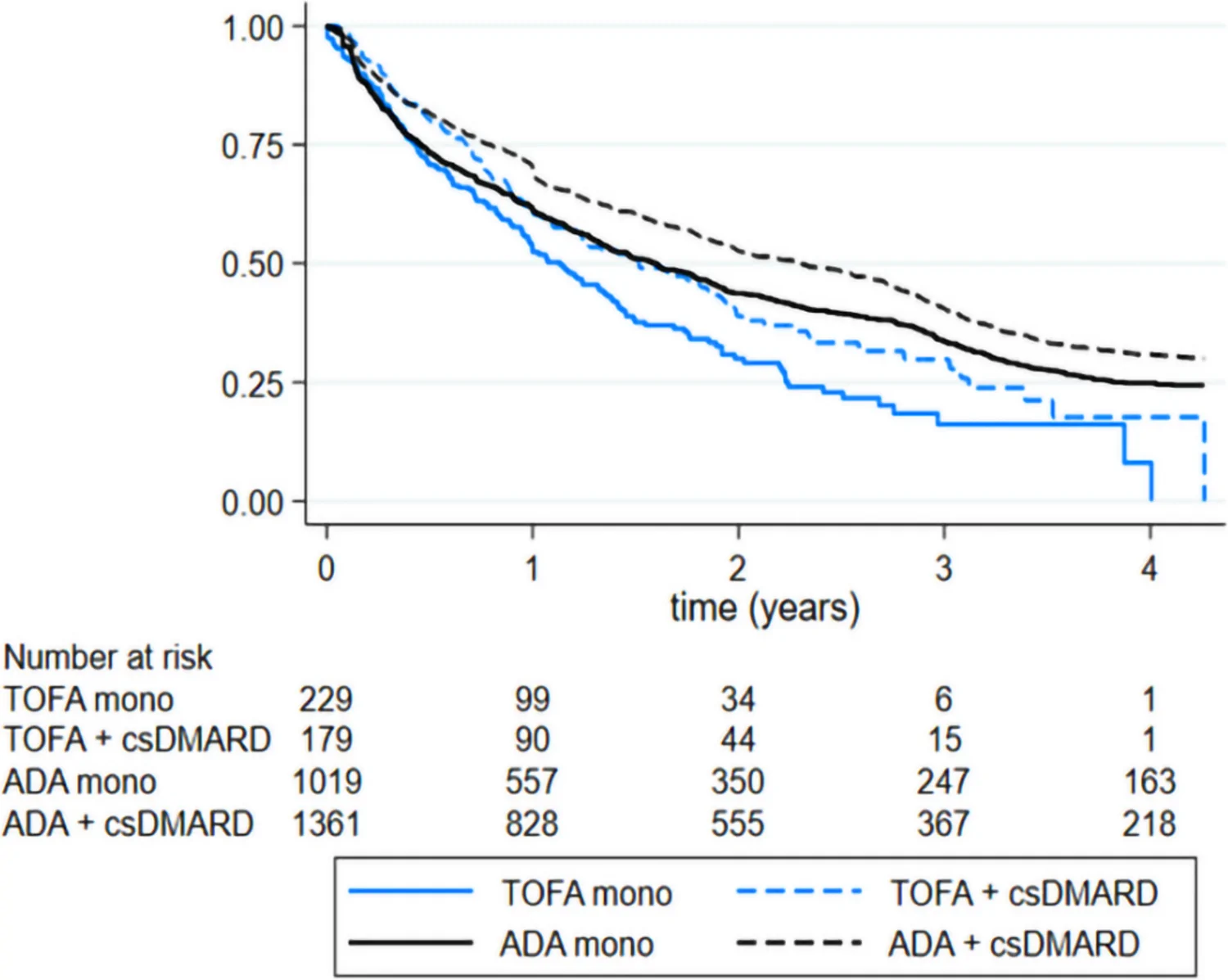
Kaplan Meier estimates of crude drug survival up to four years treatment in new initiators of tofacitinib and adalimumab in mono- and combination therapy. ADA mono, adalimumab monotherapy; ADA + csDMARD, adalimumab combination therapy; csDMARD, conventional synthetic disease-modifying antirheumatic drug; TOFA mono, tofacitinib monotherapy; TOFA + csDMARD, tofacitinib combination therapy

The crude median drug survival was 1.4, 1.8, 1.9 and 2.8 years, for tofacitinib mono-, combination- and adalimumab mono- and combination therapy, respectively (*p* < 0.001). Throughout the study period, significant higher drug persistence rates were observed for adalimumab combination therapy compared to patients treated with tofacitinib monotherapy (*p* < 0.001) (Fig. 1). Estimated drug persistence rates at 6 and 12 months were 76% (95% CI 0.69–0.81) and 58% (95% CI 0.51–0.65) in the tofacitinib monotherapy group, and 82% (95% CI 0.75–0.87) and 65% (95% CI 0.57–0.72) in the tofacitinib combination therapy group.

Drug persistence rates at 6 and 12 months for patients treated with adalimumab monotherapy and combination therapy were 76% (95% CI 0.73–0.78), 65% (95% CI 0.62–0.68) and 83% (95% CI 0.81–0.85), 72% (95% CI 0.69–0.75), respectively. The proportional hazards assumption was met (on the basis of Schoenfeld residuals) for the adjusted cox model with time to drug discontinuation as dependent variable and treatment group as independent variable (*p* = 0.358) indicating that for all treatment groups the relative risk for drug discontinuation was constant over time.

After adjusting for possible confounders, Cox regression analyses demonstrated a similar overall risk of drug discontinuation for tofacitinib combination therapy compared to tofacitinib monotherapy: HR 1.22 (95% CI 0.96–1.54, *p* = 0.11).

The overall risk of drug discontinuation with adalimumab monotherapy did not differ significantly from that of tofacitinib monotherapy (HR 0.89, 95% CI 0.73–1.09, *p* = 0.27). However, compared to tofacitinib monotherapy, drug survival was higher with adalimumab combination therapy (HR 0.78, 95% CI 0.63–0.96, *p* = 0.02) (Supplementary Table S1.4). However, this finding should be interpreted in the context of potential residual confounding and incomplete data on prior b/tsDMARD use.

The comparative unadjusted analyses and sensitivity analyses not taking a DAS ≥ 2.6 into account are shown in Supplementary Tables S1.4 and S1.5 (online resource) and provided similar results.

Further analyses on the interaction between ACPA status or age with treatment group showed no modified effect of treatment on drug survival for ACPA status (Supplementary Table S1.6, online resource). However, age significantly modified the effect of treatment on drug survival, indicating that the risk of drug discontinuation increases with increase in age. Including an interaction term between age (continuous) and treatment group resulted in an increased HR of 1.02 (95% CI 0.99–1.04, *p* = 0.06), 1.01 (95% CI 0.99–1.03, *p* = 0.05) and 1.02 (95% CI 1.00–1.03, *p* = 0.02) for tofacitinib combination therapy, adalimumab mono- and adalimumab combination therapy, respectively (Supplementary Table S1.8, online resource). Unadjusted and sensitivity analyses not taking a DAS ≥ 2.6 into account show a similar direction of results and are reported in Supplementary Tables S1.6–S1.9 (online resource). After adjusting for differences in baseline hazards for elderly and younger patients (age groups ≥ 65 and < 55), no difference in the risk of drug discontinuation was observed between tofacitinib monotherapy, tofacitinib combination therapy and adalimumab monotherapy (tofacitinib combination therapy HR 1.19, 95% CI 0.94–1.52, *p* = 0.14, adalimumab monotherapy HR 0.88, 95% CI 0.71–1.08, *p* = 0.21) in both age groups (Table 2).

**Table 2:** Results of Cox regression model with time to drug discontinuation as dependent variable and treatment group as independent variable, after adjusting for differing baseline hazards by age group (≥ 65 and < 55 years old). Drug discontinuation is defined by DAS28 > 2.6 (no remission)

	Drug survival					
		Unadjusted			Adjusted	
	*HR*	*95% CI*	*P* value	*HR*	*95% CI*	*P* value
TOFA mono	*Reference group*					
TOFA + csDMARD	0.88	0.71 1.09	0.25	1.19	0.94 1.52	0.14
**ADA mono**	**0.68**	**0.57 0.81**	** < 0.001**	0.88	0.71 1.08	0.21
**ADA + csDMARD**	**0.56**	**0.48 0.67**	** < 0.001**	**0.76**	**0.62 0.94**	**0.01**

Drug discontinuation is defined by stop date of tofacitinib/adalimumab in combination with DAS ≥ 2.6 (no remission)

Adjusted for confounders: sex, ACPA +, smoking status, baseline DAS28, follow-up time and previous b/tsDMARD use

*ADA mono*, adalimumab monotherapy; *95% CI*, 95% confidence interval; *csDMARD*, conventional synthetic disease-modifying antirheumatic drug; *DAS28*, disease activity score assessed using 28 joints; *HR*, hazard ratio; *TOFA*, tofacitinib

After adjusting for differences in baseline hazards for elderly and younger patients, patients treated with adalimumab combination therapy still had a significantly lower risk of drug discontinuation compared to tofacitinib monotherapy (pairwise HR for adalimumab combination therapy vs tofacitinib monotherapy 0.76, 95% CI 0.62–0.94, *p* = 0.01 in both age groups (Table 2).

### Sensitivity analysis

To assess the influence of correlation within patients, a sensitivity analysis was performed only including the duration of the first assigned b/tsDMARD treatment (adalimumab/tofacitinib). Overall, Cox proportional hazard analyses assessing the effect of treatment on drug survival in this subgroup revealed similar direction of results as the primary analyses. Results are presented in Supplementary Tables S1.11 through S1.16 (online resource). Compared to tofacitinib monotherapy, the risk of drug discontinuation was lower for both adalimumab monotherapy (aHR 0.78, 95% CI 0.63; 0.98, *p* = 0.030) and adalimumab combination therapy (aHR 0.69, 95% CI 0.55; 0.86, *p* = 0.001). To account for the effect of availability of newer treatment options on decisions to stop adalimumab treatment, all primary analyses were repeated only including patients who had initiated adalimumab after 2009 and by only including patients who had initiated adalimumab after 2012. These sensitivity analyses also revealed similar direction of results as the primary analyses. Results are presented in Supplementary Tables S1.17 through S1.28 (online resource). Because line of treatment can possibly affect treatment effectiveness and drug survival, we performed a third sensitivity analyses by only including patients who had not previously used a b/tsDMARD. The percentage of missingness for prior b/tsDMARD use was 39, 31 and 1% in the treatment groups tofacitinib mono-, tofacitinib combination therapy and adalimumab monotherapy, respectively. Again, Cox proportional hazard analyses only looking at tofacitinib/adalimumab users without prior b/tsDMARD use revealed similar direction of results as the primary analyses, where both adalimumab treatment groups had a significant lower risk of drug discontinuation compared to tofacitinib monotherapy (adalimumab monotherapy aHR 0.70, 95% CI 0.52; 0.95, *p* = 0.021 and adalimumab combination therapy aHR 0.61, 95% CI 0.45; 0.83, *p* = 0.001) (Supplementary Table S1.29, online resource). The sensitivity analyses performed on imputed datasets in which the assumed number of previous used b/tsDMARDs was zero in patients initiating tofacitinib revealed a similar direction of results as the primary analyses (Supplementary Tables S2.1 through S2.6, online resource). However, the sensitivity analyses performed on imputed datasets in which the assumed number of previous used b/tsDMARDs was at least one or more than two, revealed different results. Namely, for both assumptions, compared to tofacitinib monotherapy, the adjusted risk of drug discontinuation was not significantly different across all treatment groups (Supplementary Tables S3.1 through S3.6 and S4.1 through S4.6, online resource). Data on prior b/tsDMARD use were frequently missing in the tofacitinib group, which may have contributed to the loss of statistical significance in the sensitivity analyses.

## Discussion

In this real-world study on the comparative effectiveness of tofacitinib monotherapy to tofacitinib in combination with csDMARDs, and adalimumab in mono- and combination therapy, we found similar drug survival for tofacitinib mono- and combination therapy, suggesting that the use of concomitant csDMARDs may have limited added benefit to maintain effectiveness. Baseline ACPA status did not influence drug survival. However, older age at treatment start was associated with a higher risk of drug discontinuation. Adalimumab combination therapy showed slightly better drug survival than tofacitinib monotherapy, although effect sizes were small and likely not clinically relevant. This finding should be interpreted with caution due to the potential for residual confounding and missingness in prior b/tsDMARD data.

Tofacitinib has proven effective in RA in both in mono- and combination therapy, in clinical trials [21, 22]. In the ORAL strategy trial, which investigated the comparative effectiveness of tofacitinib monotherapy, tofacitinib plus MTX and adalimumab plus MTX in MTX-inadequate responders, tofacitinib did not demonstrate non-inferiority to combination therapy or adalimumab plus MTX [2]. However, efficacy of a drug in clinical trials may differ from the effectiveness of that same drug in daily practice, which is known as the efficacy-effectiveness gap [20]. Therefore, real world data may complement RCT data to help determine the best course of treatment after csDMARD failure. Few real world studies have compared tofacitinib mono- and combination therapy. Data from the CorEvitas registry found no significances in differences in treatment effectiveness, measured in modified ACR 20% response, pain and CDAI low disease activity at six months between tofacitinib monotherapy and combination therapy, nor compared to TNFi combination therapy in third and fourth lines (prior use of at least one csDMARD and one or two bDMARDs) [14]. Differences may reflect baseline disease activity, which was somewhat lower in our study than in the Corrona registry. A study by Finckh et al. reported higher retention with tofacitinib vs TNFi (aHR 1.29 (95% CI 1.14–1.47)), which was not influenced by csDMARD co-treatment [12]. However, whether tofacitinib monotherapy is as effective as each individual TNFi in combination with a csDMARD cannot be concluded from these studies. Another study directly compared adalimumab with tofacitinib in real-world data, using an intention to treat analysis including stable balancing weights to account for differences in baseline characteristics, and found no significant difference in DAS28-CRP reduction at 9 months [23]. However, this study did not take into account whether patients did or did not use concomitant csDMARDs.

In the current study, we aimed to extend real-world evidence on the comparative effectiveness of tofacitinib mono- and combination therapy, with a focus on serological and age subgroups often underrepresented in clinical trials. In daily clinical practice, the proportion of patients who are ACPA-negative is likely to be greater than in RCTs. In tofacitinib users, older age has been related to worse drug survival due to toxic adverse events [13, 24–26]. We also observed higher rates of drug discontinuation with older age, for both tofacitinib and adalimumab treatment. The effect of age on drug survival was assessed exploratorily. Although drug survival can be seen as a proxy for long term treatment effectiveness and safety, the observed associations should not be interpreted as solely reflecting treatment efficacy, as discontinuation may be influenced by factors beyond effectiveness, including safety, physician preference and comorbidity burden. Older age is related to a higher risk of treatment associated harm and a greater burden of comorbidities, making safety issues a likely contributor to discontinuation [27–32]. Although we adjusted for disease activity, adverse events may still contribute to failure. As safety events were not systematically captured or compared in this study, the association between age and treatment discontinuation cannot be fully attributed to differences in treatment efficacy. The impact of ACPA status on drug survival remains unclear, with conflicting prior results. While previous long term follow-up and post hoc studies of RCT’s show better ACR20/50/70 responses and drug survival with tofacitinib in ACPA-positive patients, an observational study investigating the comparative three year drug retention of tofacitinib and TNFi found a positive association between ACPA-negative status and drug survival [26, 33, 34]. Consistent with our findings, Bilgin et al. observed no effect of ACPA status on drug survival [13]. However, this result should be interpreted with caution given limitations in data completeness and variability in ACPA testing across centres, which may have affected the reliability of this subgroup analysis.

To our knowledge, this is the first real world study in which direct comparison of tofacitinib and adalimumab treatment effectiveness, in mono- and combination therapy, was performed in specific RA subgroups using real world clinical data. Other strengths include multicenter data and a relatively large study population. Nonetheless, several limitations should be acknowledged. First, this study holds limitations related to the retrospective nature of observational data, such as missingness, non-standardized data collection, uncertainty in treatment adherence and nonrandomized treatment assignment. Furthermore, difference in baseline disease characteristics may skew comparisons between treatment groups. However, interestingly, the proportion of patients using mono- and combination therapy were similar within tofacitinib and adalimumab treatment groups. To address missingness in baseline covariates, multiple imputation was applied, enabling inclusion of all available patients in the analyses. However, the relatively high proportion of missing data for certain variables, such as disease activity (DAS28), may have led to bias in the imputed values.

Second, although we adjusted for baseline differences between treatment groups, including age, disease stage, and treatment line, residual channelling bias may persist and the results should therefore be interpreted with caution. Any observed treatment benefits should be considered in the context of these underlying differences. Channeling biases may also persist because of unmeasured confounders. Possible confounders which may influence drug survival and treatment decisions, such as BMI, disease duration and comorbidities were not taken into account because these data were not available. Similarly, due the unreliable registration of the dosage of csDMARDs and the type and dosage of glucocorticoid use, these were not taken into account, although they may influence drug survival and treatment decisions.

We performed all analyses adjusting for known and available confounders. A specific point of consideration raised by the comparative design is the imbalance in prior treatment history between the tofacitinib and adalimumab groups. Patients receiving tofacitinib were more frequently treated in later lines of therapy, which reflects real-world prescribing patterns in which newer agents are often reserved for patients with prior treatment failure. As line of therapy is a known determinant of drug survival, with earlier-line treatments typically showing higher persistence, this imbalance may influence the observed outcomes and introduces the possibility of channeling bias. To account for this, we adjusted all analyses for available confounders using multivariable Cox regression models. In addition, we performed sensitivity analyses restricting the adalimumab cohort to patients initiating treatment after 2009, thereby addressing temporal differences in treatment availability and positioning. These analyses yielded results consistent with the primary findings, supporting the robustness of the observed effects. However, as prior treatment history was incompletely captured, particularly in the tofacitinib group, this remains an important consideration, and residual confounding cannot be fully excluded. In addition, despite consistent findings across calendar years, the main comparisons should be interpreted cautiously given changes in treatment strategies over the study period.

A sensitivity analysis examining alternative assumptions for missing prior biologic/tsDMARD exposure demonstrated that the statistical significance of the treatment comparison was sensitive to the handling of these missing data. Specifically, when all tofacitinib users with missing treatment history were assumed to have received ≥ 1 prior biologic/tsDMARD, no significant differences between treatment groups were observed. This finding suggests that uncertainty regarding prior treatment exposure may influence the strength of the observed association and should be considered when interpreting the primary results.

Third, the number of patients receiving treatment with tofacitinib and available follow-up time were considerably lower than for adalimumab. We have therefore censored follow-up duration of patients on adalimumab to align more closely with the shorter observation period for tofacitinib. While this approach improves comparability, it may have caused an over- or underestimation of the observed effect as data on the time at risk for patients treated for considerably longer periods of time is lost, introducing a trend bias. In addition, assumptions regarding treatment continuation in the presence of missing stop dates were applied uniformly across groups to minimize bias. Finally, adverse events and safety outcomes could not be evaluated in relation to drug survival due to data unavailability. Furthermore, recent regulatory restrictions on JAKi use, advising to restrict prescription of JAKi to patients under 65 years, without previous history of or risk factors for cardiovascular diseases and malignancies, may limit generalizability of these findings to this patient population.

In conclusion, the results of this study suggest that real-world drug survival, evaluated as an indirect measure of treatment persistence, did not differ significantly between tofacitinib monotherapy and combination therapy across ACPA subgroups. We found that older age may influence drug survival of both tofacitinib and adalimumab. However, especially in older age groups drug survival may be influenced by factors beyond effectiveness, including safety, patient and physician preferences and comorbidity burden. Comparisons involving adalimumab should be interpreted with caution due to residual confounding and missing data, particularly regarding prior b/tsDMARD exposure. Further research is needed to better understand the observed association between age and drug survival.

## Supplementary Information

Below is the link to the electronic supplementary material.

ESM1 (DOCX 208 KB)

ESM2 (DOCX 65.8 KB)

ESM3 (DOCX 33.5 KB)

## Data Availability

The anonymized dataset used and analyzed during the current study are available from the corresponding author on reasonable request.
