# Supplementary material for: Real-world effectiveness of tofacitinib in monotherapy or in combination with csDMARDs in patients with rheumatoid arthritis: A collaboration between Dutch registries (TOFADUDAP study)
Source: Clin Rheumatol. 2026 Aug 19;45(10):6071–80. doi: 10.1007/s10067-026-08362-x (PMC13582166; doi:10.1007/s10067-026-08362-x)
Supplement: Supplementary file 1 [file 10067_2026_8362_MOESM1_ESM.docx]

# **SUPPLEMENTARY INFORMATION 1**

## **Fig. S1.1 Study flow chart**

## Table S1.1 Baseline characteristics per hospital.

|  | **HMC** | **MCL** | **LUMC** | **MST** | **RZWNL** | **SMK** |
| --- | --- | --- | --- | --- | --- | --- |
| N, % of total | 59 (2) | 395 (15) | 513 (19) | 423 (16) | 620 (23) | 674 (25) |
| Female, % | 83 | 63 | 73 | 66 | 69 | 69 |
| *Missingness, %* | - | - | *1* | - | - | - |
| TOFA mono, N (% of total) | 18 (27) | 12(3) | 5 (1) | 15 (3) | 141 (22) | 38 (6) |
| TOFA + csDMARD, N (% of total) | 28 (43) | 51 (12) | 14 (2) | 4 (1) | 73 (11) | 9 (1) |
| ADA mono, N (% of total) | 4 (6) | 116 (28) | 82 (16) | 283 (65) | 253 (39) | 281 (41) |
| ADA + csDMARD, N (% of total) | 16 (24) | 241 (57) | 422 (81) | 136 (31) | 185 (28) | 361 (52) |
| Age at start tofa, mean (SD) | 47 (12) | 56 (15) | 59 (11) | 59 (10) | 62 (13) | 57 (13) |
| *Missingness, %* | *-* | *-* | - | *-* | *-* |  |
| Age at start ada, mean (SD) | 42 (13) | 60 (13) | 56 (12) | 55 (14) | 61 (13) | 57 (14) |
| *Missingness, %* | *-* | *-* | *1* | *-* | *-* | *-* |
| Number of previous b/tsDMARDs before start of tofa/ada, % |  |  |  |  |  |  |
| *0* | 58 (54 Tofa, 65 Ada) | 83 (41 Tofa, 90 Ada) | 79 (16 Tofa, 81 Ada) | 77 (5 Tofa, 81 Ada) | 71 (20 Tofa, 97 Ada) | 75 (21 Tofa, 79 Ada) |
| *1* | 24 (26 Tofa, 20 Ada) | 13 (33 Tofa, 9 Ada) | 15 (5 Tofa, 16 Ada) | 15 (26 Tofa, 14 Ada) | 4 (9 Tofa, 2 Ada) | 18 (9 Tofa, 19 Ada) |
| *≥2* | 18 (20 Tofa, 15 Ada) | 5 (25 Tofa, 1 Ada) | 6 (79 Tofa, 3 Ada) | 7 (68 Tofa, 4 Ada) | 2 (4 Tofa, 1 Ada) | 7 (70 Tofa, 2 Ada) |
| DAS28 at start of tofa, mean (SD) | 3.0 (1.2) | 3.8 (1.2) | 4.3 (1.1) | 3.9 (1.3) | 3.1 (1.3) | 3.5 (1.7) |
| *Missingness, %* | *76* | *52* | *26* | *11* | *67* | *72* |
| DAS28 at start of ada, mean (SD) | 3.5 (1.8) | 3.3 (1.3) | 3.7 (1.5) | 4.1 (1.5) | 2.6 (1.2) | 4.2 (1.5) |
| *Missingness, %* | *70* | *65* | *22* | *8* | *49* | *86* |
| RF positive, % | 74 | 100 | 85 | 84 | 58 | 66 |
| *Missingness, %* | *-* | *-* | *-* | *-* | *-* | *-* |
| ACPA positive, % | 64 | 22 | 62 | 51 | 31 | 67 |
| *Missingness, %* | *33* | *61* | *27* | *32* | *45* | *1* |
| Smoker/has ever smoked, % | 9 | 65 | 23 | 31 | 12 | 27 |
| *Missingness, %* | *71* | *7* | *58* | *45* | *81* | *48* |
| Ada, adalimumab; ADA mono, adalimumab monotherapy; 95% CI, 95% confidence interval; b/tsDMARDs, biologic/targeted synthetic disease-modifying antirheumatic drugs; csDMARD, conventional synthetic disease-modifying antirheumatic drug; DAS28, disease activity score assessed using 28 joints, in case of missing values a new DAS28 score was calculated based on the individual components or the DAS28 score within a window of 4 weeks before baseline was carried forward; NA, Not Available; RF+, Rheumatoid Factor positive; Smoker, patients who were smokers or who had ever smoked; TOFA, tofacitinib; tofa, tofacitinib | | | | | | |

## Table S1.2 Baseline characteristics for tofacitinib and adalimumab mono- and combination treatment groups, stratified by ACPA status.

|  | **TOFA mono** | |  | **TOFA + csDMARD** | |  | **ADA mono** | |  | **ADA + csDMARD** | |  |
| --- | --- | --- | --- | --- | --- | --- | --- | --- | --- | --- | --- | --- |
|  | **ACPA +** | **ACPA -** | ***P value*** | **ACPA +** | **ACPA -** | ***P value*** | **ACPA +** | **ACPA -** | ***P value*** | **ACPA +** | **ACPA -** | ***P value*** |
| N, % of total | 127  (56) | 75  (33) |  | 89  (50) | 40  (22) |  | 444  (44) | 218 (21) |  | 689  (51) | 247 (18) |  |
| Female, % | 72 | 72 | *0.96* | 78 | 73 | *0.54* | 73 | 74 | *0.79* | 68 | 68 | *0.95* |
| *Missingness, %* | *0* | *0* |  | *0* | *0* |  | *0.2* | *0* |  | *0.3* | *0.4* |  |
| Age at start tofa, mean (SD) | 61  (13) | 61  (14) | *0.81* | 56  (14) | 57  (13) | *0.76* | NA | NA |  | NA | NA |  |
| *Missingness, %* | *0* | *0* |  | *0* | *0* |  |  |  |  |  |  |  |
| Age at start ada, mean (SD) | NA | NA |  | NA | NA |  | 58  (13) | 55  (15) | ***0.01*** | 57  (13) | 56  (14) | *0.19* |
| *Missingness, %* |  |  |  |  |  |  | *0* | *0* |  | *0* | *0* |  |
| Number of previous b/tsDMARDs before start of tofa/ada, % |  |  | *0.65* |  |  | *0.95* |  |  | *0.33* |  |  | ***0.04*** |
| *0* | 29 | 19 |  | 19 | 18 |  | 73 | 79 |  | 85 | 89 |  |
| *1* | 9 | 8 |  | 17 | 17 |  | 21 | 16 |  | 13 | 11 |  |
| *≥2* | 30 | 15 |  | 22 | 25 |  | 4 | 3 |  | 2 | 0 |  |
| *Missingness, %* | *32* | *58* |  | *42* | *40* |  | *2* | *1* |  | *-* | *-* |  |
| DAS28 at start of tofa/ada, mean (SD) | 3.5  (1.4) | 2.9  (1.3) | *0.12* | 3.5  (1.3) | 3.6 (1.2) | *0.78* | 4.0  (1.4) | 3.8 (1.6) | *0.21* | 3.8  (1.5) | 3.9 (1.3) | *0.39* |
| *Missingness, %* | *63* | *73* |  | *55* | *63* |  | *51* | *55* |  | *50* | *60* |  |
| RF positivity, % | 54 | 5 | ***<0.001*** | 57 | 23 | ***<0.001*** | 84 | 34 | ***<0.001*** | 85 | 27 | ***<0.001*** |
| *Missingness, %* | *37* | *49* |  | *39* | *45* |  | *9* | *8* |  | *7* | *17* |  |
| Current/ever smoker, % | 19 | 19 | *0.16* | 24 | 23 | *0.58* | 27 | 28 | *0.90* | 32 | 34 | *0.82* |
| *Missingness, %* | *71* | *65* |  | *62* | *53* |  | *52* | *48* |  | *45* | *41* |  |
| *ada, adalimumab; csDMARD, conventional synthetic disease-modifying antirheumatic drug* (*i.e. Methotrexate, Sulfasalazine, Leflunomide, Hydroxychloroquine, Azathioprine, Ciclosporine, Cyclophosphamide, Penicillamine, Aurothiomalaat, Bucillamine, Mizorbine, Tacrolimus, Auranofine*); DAS28, disease activity score assessed using 28 joints, in case of missing values a new DAS28 score was calculated based on the individual components or the DAS28 score within a window of 4 weeks before baseline was carried forward; *NA, not applicable; tofa, tofacitinib* | | | | | | | | | | | | |

## Table S1.3 Baseline characteristics for tofacitinib and adalimumab mono- and combination treatment groups, stratified by age (≥65 vs <55 years old)

|  | **TOFA mono** | |  | **TOFA + csDMARD** | |  | **ADA mono** | |  | **ADA + csDMARD** | |  |
| --- | --- | --- | --- | --- | --- | --- | --- | --- | --- | --- | --- | --- |
|  | **<55 years old** | **≥65 years old** | ***P***  ***value*** | **<55 years old** | **≥65 years old** | ***P***  ***value*** | **<55 years old** | **≥65 years old** | ***P value*** | **<55 years old** | **≥65 years old** | ***P value*** |
| N, % of total | 71 (31) | 103 (45) |  | 83 (46) | 53 (29) |  | 406  (40) | 330  (32) |  | 512  (38) | 444  (33) |  |
| Female, % | 73 | 73 | *0.95* | 84 | 72 | *0.08* | 71 | 67 | *0.21* | 72 | 66 | *0.06* |
| *Missingness, %* | *0* | *0* |  | *0* | *0* |  | *0* | *0* |  | *0.4* | *0.2* |  |
| Number of previous b/tsDMARDs before start of tofa/ada, % |  |  |  |  |  |  |  |  |  |  |  |  |
| *0* | 30 | 27 | *0.79* | 34 | 25 | *0.90* | 82 | 78 | *0.29* | 87 | 89 | *0.63* |
| *1* | 14 | 13 |  | 20 | 15 |  | 15 | 17 |  | 11 | 10 |  |
| *≥2* | 25 | 17 |  | 23 | 13 |  | 2 | 4 |  | 2 | 1 |  |
| *Missingness, %* | *31* | *43* |  | *23* | *47* |  | *1* | *1* |  | *-* | *-* |  |
| *DAS28 at start ada/tofa, mean (SD) | 3.5  (1.3) | 3.3  (1.5) | *0.58* | 3.6  (1.2) | 3.3  (1.3) | *0.46* | 3.5  (1.5) | 3.7  (1.6) | *0.38* | 3.5  (1.6) | 3.9  (1.4) | ***0.01*** |
| *Missingness, %* | *65* | *63* |  | *65* | *49* |  | 48 | 50 |  | 47 | 49 |  |
| ACPA positivity, % | 54 | 56 | *0.84* | 49 | 53 | *0.81* | 44 | 43 | *0.15* | 54 | 47 | *0.46* |
| *Missingness, %* | *16* | *9* |  | *29* | *26* |  | *31* | *39* |  | *26* | *37* |  |
| RF positivity, % | 34 | 27 | *0.59* | 35 | 47 | *0.17* | 48 | 46 | *0.32* | 55 | 50 | *0.23* |
| *Missingness, %* | *45* | *52* |  | *52* | *45* |  | 37 | 39 |  | 33 | 37 |  |
| Current/ever smoker, % | 13 | 18 | *0.63* | 22 | 45 | ***0.00*** | 27 | 23 | ***0.00*** | 25 | 31 | ***0.00*** |
| *Missingness, %* | *76* | *66* |  | *51* | *47* |  | 51 | 56 |  | 49 | 49 |  |
| ada, adalimumab; csDMARD, conventional synthetic disease-modifying antirheumatic drug (i.e. Methotrexate, Sulfasalazine, Leflunomide, Hydroxychloroquine, Azathioprine, Ciclosporine, Cyclophosphamide, Penicillamine, Aurothiomalaat, Bucillamine, Mizorbine, Tacrolimus, Auranofine); DAS28, disease activity score assessed using 28 joints, in case of missing values a new DAS28 score was calculated based on the individual components or the DAS28 score within a window of 4 weeks before baseline was carried forward; NA, not applicable; tofa, tofacitinib | | | | | | | | | | | | |

## **DRUG SURVIVAL**

## Table S1.4 Results of Cox regression model with the time to drug discontinuation as dependent variable and treatment group as independent variable. Drug discontinuation is defined by DAS28≥2.6 (no remission)^a^

|  | **Drug survival** | | | | |  |
| --- | --- | --- | --- | --- | --- | --- |
|  |  | **Unadjusted** |  |  | **Adjusted*** |  |
|  | ***HR*** | ***95% CI*** | ***P value*** | ***HR*** | ***95% CI*** | ***P value*** |
| TOFA mono | *Reference group* |  |  |  |  |  |
| TOFA + csDMARD | 0.89 | 0.72 1.11 | 0.29 | 1.22 | 0.96 1.54 | 0.11 |
| **ADA mono** | **0.69** | **0.58 0.82** | **<0.001** | 0.89 | 0.73 1.09 | 0.27 |
| **ADA + csDMARD** | **0.56** | **0.48 0.67** | **<0.001** | **0.78** | **0.63 0.96** | **0.02** |
| ^a^Drug discontinuation is defined by stop date of tofacitinib/adalimumab in combination with DAS≥2.6 (no remission)  *Adjusted for confounders: age at start TOFA/ADA, sex, ACPA +, smoking status, baseline DAS28, follow up time, previous b/tsDMARD use  ADA mono, adalimumab monotherapy; 95% CI, 95% confidence interval; csDMARD, conventional synthetic disease-modifying antirheumatic drug; DAS28, disease activity score assessed using 28 joints; HR, hazard ratio; TOFA, tofacitinib. | | | | | | |

## Table S1.5 Results of Cox regression model with the time to drug discontinuation^a^ as dependent variable and treatment group as independent variable

|  | **Drug survival** | | | | |  |
| --- | --- | --- | --- | --- | --- | --- |
|  |  | **Unadjusted** |  |  | **Adjusted*** |  |
|  | ***HR*** | ***95% CI*** | ***P value*** | ***HR*** | ***95% CI*** | ***P value*** |
| TOFA mono | *Reference group* |  |  |  |  |  |
| TOFA + csDMARD | 0.84 | 0.67 1.03 | 0.09 | 1.15 | 0.92 1.44 | 0.22 |
| **ADA mono** | **0.69** | **0.59 0.80** | **<0.001** | 0.87 | 0.72 1.04 | 0.13 |
| **ADA + csDMARD** | **0.57** | **0.48 0.66** | **<0.001** | **0.77** | **0.64 0.93** | **0.01** |
| ^a^Drug discontinuation is defined by stop date of tofacitinib/adalimumab  *Adjusted for confounders: age at start TOFA/ADA, sex, ACPA +, smoking status, baseline DAS28, follow up time, previous b/tsDMARD use  ADA mono, adalimumab monotherapy; 95% CI, 95% confidence interval; csDMARD, conventional synthetic disease-modifying antirheumatic drug; DAS28, disease activity score assessed using 28 joints; HR, hazard ratio; TOFA, tofacitinib. | | | | | | |

## Table S1.6 Results of Cox regression model with the time to drug discontinuation as dependent variable and treatment group as independent variable. Including an interaction term between treatment group and ACPA status^b^. Drug discontinuation is defined by DAS28≥2.6 (no remission)^a^

|  | **Drug survival** | | | | |  |
| --- | --- | --- | --- | --- | --- | --- |
|  |  | **Unadjusted** |  |  | **Adjusted*** |  |
|  | ***HR*** | ***95% CI*** | ***P value*** | ***HR*** | ***95% CI*** | ***P value*** |
| TOFA mono #ACPA | *Reference group* |  |  |  |  |  |
| TOFA + csDMARD #ACPA | 0.94 | 0.54 1.64 | 0.83 | 0.86 | 0.51 1.48 | 0.59 |
| ADA mono #ACPA | 0.87 | 0.59 1.27 | 0.46 | 0.88 | 0.59 1.31 | 0.51 |
| ADA + csDMARD #ACPA | 0.75 | 0.52 1.09 | 0.13 | 0.79 | 0.53 1.18 | 0.24 |
| ^a^Drug discontinuation is defined by stop date of tofacitinib/adalimumab in combination with DAS≥2.6 (no remission)  ^b^ACPA - is the reference  *Adjusted for confounders: age at start TOFA/ADA, sex, smoking status, baseline DAS28, follow up time, previous b/tsDMARD use  ADA mono, adalimumab monotherapy; 95% CI, 95% confidence interval; csDMARD, conventional synthetic disease-modifying antirheumatic drug; DAS28, disease activity score assessed using 28 joints; HR, hazard ratio; TOFA, tofacitinib. | | | | | | |

## Table S1.7 Results of Cox regression model with the time to drug discontinuation^a^ as dependent variable and treatment group as independent variable. Including an interaction term between treatment group and ACPA status^b^

|  | **Drug survival** | | | | |  |
| --- | --- | --- | --- | --- | --- | --- |
|  |  | **Unadjusted** |  |  | **Adjusted*** |  |
|  | ***HR*** | ***95% CI*** | ***P value*** | ***HR*** | ***95% CI*** | ***P value*** |
| TOFA mono #ACPA | *Reference group* |  |  |  |  |  |
| TOFA + csDMARD #ACPA | 0.90 | 0.53 1.54 | 0.71 | 0.81 | 0.49 1.32 | 0.39 |
| ADA mono #ACPA | 0.94 | 0.67 1.34 | 0.75 | 0.93 | 0.65 1.32 | 0.67 |
| ADA + csDMARD #ACPA | 0.83 | 0.59 1.17 | 0.28 | 0.84 | 0.59 1.19 | 0.32 |
| ^a^Drug discontinuation is defined by stop date of tofacitinib/adalimumab  ^b^ACPA - is the reference  *Adjusted for confounders: age at start TOFA/ADA, sex, smoking status, baseline DAS28, follow up time, previous b/tsDMARD use  ADA mono, adalimumab monotherapy; 95% CI, 95% confidence interval; csDMARD, conventional synthetic disease-modifying antirheumatic drug; DAS28, disease activity score assessed using 28 joints; HR, hazard ratio; TOFA, tofacitinib. | | | | | | |

## Table S1.8 Results of Cox regression model with the time to drug discontinuation as dependent variable and treatment group as independent variable. Including an interaction term between treatment group and age^a^. Drug discontinuation is defined by DAS28≥2.6 (no remission)^b^

|  | **Drug survival** | | | | |  |
| --- | --- | --- | --- | --- | --- | --- |
|  |  | **Unadjusted** |  |  | **Adjusted*** |  |
|  | ***HR*** | ***95% CI*** | ***P value*** | ***HR*** | ***95% CI*** | ***P value*** |
| TOFA mono #age | *Reference group* |  |  |  |  |  |
| **TOFA + csDMARD #age** | **1.01** | **0.99 1.03** | **0.09** | **1.02** | **0.99 1.04** | **0.06** |
| **ADA mono #age** | **1.02** | **1.00 1.03** | **0.01** | **1.01** | **0.99 1.03** | **0.05** |
| **ADA + csDMARD #age** | **1.02** | **1.01 1.03** | **0.01** | **1.02** | **1.00 1.03** | **0.02** |
| ^a^Age is continuous  ^b^Drug discontinuation is defined by stop date of tofacitinib/adalimumab in combination with DAS≥2.6 (no remission)  *Adjusted for confounders: sex, ACPA +, smoking status, baseline DAS28, follow up time, previous b/tsDMARD use  ADA mono, adalimumab monotherapy; 95% CI, 95% confidence interval; csDMARD, conventional synthetic disease-modifying antirheumatic drug; DAS28, disease activity score assessed using 28 joints; HR, hazard ratio; TOFA, tofacitinib. | | | | | | |

## Table S1.9 Results of Cox regression model with the time to drug discontinuation^a^ as dependent variable and treatment group as independent variable. Including an interaction term between treatment group and age^b^

|  | **Drug survival** | | | | |  |
| --- | --- | --- | --- | --- | --- | --- |
|  |  | **Unadjusted** |  |  | **Adjusted*** |  |
|  | ***HR*** | ***95% CI*** | ***P value*** | ***HR*** | ***95% CI*** | ***P value*** |
| **TOFA mono #age** | *Reference group* |  |  |  |  |  |
| TOFA + csDMARD #age | 1.01 | 0.99 1.03 | 0.11 | 1.02 | 0.99 1.03 | 0.11 |
| ADA mono #age | 1.01 | 0.99 1.02 | 0.13 | 1.01 | 0.99 1.02 | 0.15 |
| **ADA + csDMARD #age** | **1.01** | **0.99 1.02** | **0.09** | 1.01 | 0.99 1.02 | 0.13 |
| ^a^Drug discontinuation is defined by stop date of tofacitinib/adalimumab  ^b^Age is continuous  *Adjusted for confounders: sex, ACPA +, smoking status, baseline DAS28, follow up time, previous b/tsDMARD use  ADA mono, adalimumab monotherapy; 95% CI, 95% confidence interval; csDMARD, conventional synthetic disease-modifying antirheumatic drug; DAS28, disease activity score assessed using 28 joints; HR, hazard ratio; TOFA, tofacitinib. | | | | | | |

## Table S1.10 Results of Cox regression model with the time to drug discontinuation^a^ as dependent variable and treatment group as independent variable, after adjusting for differing baseline hazards by age group (≥65 vs <55 years old)

|  | | **Drug survival** | | | | |  |
| --- | --- | --- | --- | --- | --- | --- | --- |
|  | |  | **Unadjusted** |  |  | **Adjusted*** |  |
|  | | ***HR*** | ***95% CI*** | ***P value*** | ***HR*** | ***95% CI*** | ***P value*** |
| TOFA mono | *Reference group* | |  |  |  |  |  |
| TOFA + csDMARD | | 0.83 | 0.67 1.02 | 0.07 | 1.15 | 0.92 1.43 | 0.24 |
| **ADA mono** | | **0.68** | **0.58 0.79** | **<0.001** | 0.86 | 0.71 1.03 | 0.11 |
| **ADA + csDMARD** | | **0.56** | **0.48 0.66** | **<0.001** | **0.76** | **0.63 0.92** | **0.01** |
| ^a^Drug discontinuation is defined by stop date of tofacitinib/adalimumab  *Adjusted for confounders:, sex, ACPA +, smoking status, baseline DAS28, follow up time, previous b/tsDMARD use  ADA mono, adalimumab monotherapy; 95% CI, 95% confidence interval; csDMARD, conventional synthetic disease-modifying antirheumatic drug; DAS28, disease activity score assessed using 28 joints; HR, hazard ratio; TOFA, tofacitinib. | | | | | | | |

## **DRUG SURVIVAL - SENSITIVITY ANALYSIS**

Table S1.11 Results of Cox regression model with the time to drug discontinuation as dependent variable and treatment groups as independent variable. Only including the duration of the first assigned b/tsDMARD treatment (adalimumab/tofacitinib). Drug discontinuation is defined by DAS28≥2.6 (no remission)^a^

|  | **Drug survival** | | | | |  |
| --- | --- | --- | --- | --- | --- | --- |
|  |  | **Unadjusted** |  |  | **Adjusted*** |  |
|  | ***HR*** | ***95% CI*** | ***P value*** | ***HR*** | ***95% CI*** | ***P value*** |
| TOFA mono | *Reference group* |  |  |  |  |  |
| TOFA + csDMARD | 0.81 | 0.63 1.03 | 0.08 | 1.05 | 0.79 1.38 | 0.73 |
| **ADA mono** | **0.65** | **0.54 0.78** | **<0.001** | **0.78** | **0.63 0.98** | **0.03** |
| **ADA + csDMARD** | **0.54** | **0.45 0.64** | **<0.001** | **0.69** | **0.55 0.86** | **0.001** |
| ^a^Drug discontinuation is defined by stop date of tofacitinib/adalimumab in combination with DAS≥2.6 (no remission)  *Adjusted for confounders: age at start TOFA/ADA, sex, ACPA +, smoking status, baseline DAS28, follow up time, previous b/tsDMARD use  ADA mono, adalimumab monotherapy; 95% CI, 95% confidence interval; csDMARD, conventional synthetic disease-modifying antirheumatic drug; DAS28, disease activity score assessed using 28 joints; HR, hazard ratio; TOFA, tofacitinib. | | | | | | |

Table S1.12 Results of Cox regression model with the time to drug discontinuation^a^ as dependent variable and treatment groups as independent variable. Only including the duration of the first assigned b/tsDMARD treatment (adalimumab/tofacitinib)

|  | **Drug survival** | | | | |  |
| --- | --- | --- | --- | --- | --- | --- |
|  |  | **Unadjusted** |  |  | **Adjusted*** |  |
|  | ***HR*** | ***95% CI*** | ***P value*** | ***HR*** | ***95% CI*** | ***P value*** |
| TOFA mono | *Reference group* |  |  |  |  |  |
| TOFA + csDMARD | **0.75** | **0.59 0.94** | **0.01** | 1.00 | 0.78 1.29 | 0.99 |
| **ADA mono** | **0.63** | **0.53 0.74** | **<0.001** | **0.76** | **0.63 0.93** | **0.01** |
| **ADA + csDMARD** | **0.52** | **0.44 0.61** | **<0.001** | **0.68** | **0.56 0.82** | **<0.001** |
| ^a^Drug discontinuation is defined by stop date of tofacitinib/adalimumab  *Adjusted for confounders: age at start TOFA/ADA, sex, ACPA +, smoking status, baseline DAS28, follow up time, previous b/tsDMARD use  ADA mono, adalimumab monotherapy; 95% CI, 95% confidence interval; csDMARD, conventional synthetic disease-modifying antirheumatic drug; DAS28, disease activity score assessed using 28 joints; HR, hazard ratio; TOFA, tofacitinib. | | | | | | |

Table S1.13 Results of Cox regression model with the time to drug discontinuation as dependent variable and treatment groups as independent variable. Including an interaction term between treatment group and ACPA status^a^. Only including the duration of the first assigned b/tsDMARD treatment (adalimumab/tofacitinib). Drug discontinuation is defined by DAS28≥2.6 (no remission)^b^

|  | **Drug survival** | | | | |  |
| --- | --- | --- | --- | --- | --- | --- |
|  |  | **Unadjusted** |  |  | **Adjusted*** |  |
|  | ***HR*** | ***95% CI*** | ***P value*** | ***HR*** | ***95% CI*** | ***P value*** |
| TOFA mono #ACPA | *Reference group* |  |  |  |  |  |
| TOFA + csDMARD #ACPA | 0.73 | 0.41 1.32 | 0.29 | 0.68 | 0.37 1.25 | 0.22 |
| ADA mono #ACPA | 0.79 | 0.54 1.19 | 0.28 | 0.83 | 0.54 1.29 | 0.42 |
| ADA + csDMARD #ACPA | **0.67** | **0.45 0.99** | **0.05** | 0.74 | 0.48 1.15 | 0.18 |
| ^a^ACPA - is the reference  ^b^Drug discontinuation is defined by stop date of tofacitinib/adalimumab in combination with DAS≥2.6 (no remission)  *Adjusted for confounders: age at start TOFA/ADA, sex, smoking status, baseline DAS28, follow up time, previous b/tsDMARD use  ADA mono, adalimumab monotherapy; 95% CI, 95% confidence interval; csDMARD, conventional synthetic disease-modifying antirheumatic drug; DAS28, disease activity score assessed using 28 joints; HR, hazard ratio; TOFA, tofacitinib. | | | | | | |

Table S1.14 Results of Cox regression model with the time to drug discontinuation^a^ as dependent variable and treatment groups as independent variable. Including an interaction term between treatment group and ACPA status^b^. Only including the duration of the first assigned b/tsDMARD treatment (adalimumab/tofacitinib)

|  | **Drug survival** | | | | |  |
| --- | --- | --- | --- | --- | --- | --- |
|  |  | **Unadjusted** |  |  | **Adjusted*** |  |
|  | ***HR*** | ***95% CI*** | ***P value*** | ***HR*** | ***95% CI*** | ***P value*** |
| TOFA mono #ACPA | *Reference group* |  |  |  |  |  |
| TOFA + csDMARD #ACPA | 0.69 | 0.39 1.19 | 0.19 | 0.64 | 0.37 1.10 | 0.11 |
| ADA mono #ACPA | 0.88 | 0.61 1.27 | 0.49 | 0.88 | 0.59 1.31 | 0.54 |
| ADA + csDMARD #ACPA | 0.76 | 0.53 1.09 | 0.13 | 0.79 | 0.54 1.17 | 0.24 |
| ^a^Drug discontinuation is defined by stop date of tofacitinib/adalimumab  ^b^ACPA - is the reference  *Adjusted for confounders: age at start TOFA/ADA, sex, smoking status, baseline DAS28, follow up time, previous b/tsDMARD use  ADA mono, adalimumab monotherapy; 95% CI, 95% confidence interval; csDMARD, conventional synthetic disease-modifying antirheumatic drug; DAS28, disease activity score assessed using 28 joints; HR, hazard ratio; TOFA, tofacitinib. | | | | | | |

Table S1.15 Results of Cox regression model with the time to drug discontinuation as dependent variable and treatment groups as independent variable. Including an interaction term between treatment group and age^a^. Only including the duration of the first assigned b/tsDMARD treatment (tofacitinib/adalimumab). Drug discontinuation is defined by DAS28≥2.6 (no remission)^b^

|  | **Drug survival** | | | | |  |
| --- | --- | --- | --- | --- | --- | --- |
|  |  | **Unadjusted** |  |  | **Adjusted*** |  |
|  | ***HR*** | ***95% CI*** | ***P value*** | ***HR*** | ***95% CI*** | ***P value*** |
| TOFA mono #age | *Reference group* |  |  |  |  |  |
| **TOFA + csDMARD #age** | **1.02** | **0.99 1.04** | **0.06** | **1.01** | **0.99 1.04** | **0.06** |
| **ADA mono #age** | **1.02** | **1.00 1.03** | **0.01** | **1.01** | **0.99 1.03** | **0.07** |
| **ADA + csDMARD #age** | **1.02** | **1.01 1.035** | **0.004** | **1.02** | **1.00 1.03** | **0.03** |
| ^a^Age is continuous  ^b^Drug discontinuation is defined by stop date of tofacitinib/adalimumab in combination with DAS≥2.6 (no remission)  *Adjusted for confounders: sex, ACPA +, smoking status, baseline DAS28, follow up time, previous b/tsDMARD use  ADA mono, adalimumab monotherapy; 95% CI, 95% confidence interval; csDMARD, conventional synthetic disease-modifying antirheumatic drug; DAS28, disease activity score assessed using 28 joints; HR, hazard ratio; TOFA, tofacitinib. | | | | | | |

Table S1.16 Results of Cox regression model with the time to drug discontinuation^a^ as dependent variable and treatment groups as independent variable. Including an interaction term between treatment group and age^b^. Only including the duration of the first assigned b/tsDMARD treatment (adalimumab/tofacitinib)

|  | **Drug survival** | | | | |  |
| --- | --- | --- | --- | --- | --- | --- |
|  |  | **Unadjusted** |  |  | **Adjusted*** |  |
|  | ***HR*** | ***95% CI*** | ***P value*** | ***HR*** | ***95% CI*** | ***P value*** |
| TOFA mono #age | *Reference group* |  |  |  |  |  |
| **TOFA + csDMARD #age** | **1.02** | **1.00 1.04** | **0.05** | **1.02** | **0.99 1.04** | **0.08** |
| ADA mono #age | 1.01 | 0.99 1.02 | 0.11 | 1.01 | 0.99 1.02 | 0.19 |
| **ADA + csDMARD #age** | **1.01** | **0.99 1.025** | **0.08** | 1.01 | 0.99 1.02 | 0.18 |
| ^a^Drug discontinuation is defined by stop date of tofacitinib/adalimumab  ^b^Age is continuous  *Adjusted for confounders: sex, ACPA +, smoking status, baseline DAS28, follow up time, previous b/tsDMARD use  ADA mono, adalimumab monotherapy; 95% CI, 95% confidence interval; csDMARD, conventional synthetic disease-modifying antirheumatic drug; DAS28, disease activity score assessed using 28 joints; HR, hazard ratio; TOFA, tofacitinib. | | | | | | |

## Table S1.17 Results of Cox regression model with the time to drug discontinuation as dependent variable and treatment groups as independent variable. Only including patients who started Adalimumab>2009. Drug discontinuation is defined by DAS28≥2.6 (no remission)^a^

|  | **Drug survival** | | | | |  |
| --- | --- | --- | --- | --- | --- | --- |
|  |  | **Unadjusted** |  |  | **Adjusted*** |  |
|  | ***HR*** | ***95% CI*** | ***P value*** | ***HR*** | ***95% CI*** | ***P value*** |
| TOFA mono | *Reference group* |  |  |  |  |  |
| TOFA + csDMARD | 0.89 | 0.71 1.11 | 0.29 | 1.21 | 0.95 1.54 | 0.12 |
| **ADA mono** | **0.75** | **0.63 0.89** | **0.001** | 0.89 | 0.73 1.09 | 0.28 |
| **ADA + csDMARD** | **0.59** | **0.50 0.70** | **<0.001** | **0.75** | **0.61 0.93** | **0.01** |
| ^a^Drug discontinuation is defined by stop date of tofacitinib/adalimumab in combination with DAS≥2.6 (no remission)  *Adjusted for confounders: age at start TOFA/ADA, sex, ACPA +, smoking status, baseline DAS28, follow up time, previous b/tsDMARD use  ADA mono, adalimumab monotherapy; 95% CI, 95% confidence interval; csDMARD, conventional synthetic disease-modifying antirheumatic drug; DAS28, disease activity score assessed using 28 joints; HR, hazard ratio; TOFA, tofacitinib. | | | | | | |

## Table S1.18 Results of Cox regression model with the time to drug discontinuation^a^ as dependent variable and treatment groups as independent variable. Only including patients who started Adalimumab>2009

|  | **Drug survival** | | | | |  |
| --- | --- | --- | --- | --- | --- | --- |
|  |  | **Unadjusted** |  |  | **Adjusted*** |  |
|  | ***HR*** | ***95% CI*** | ***P value*** | ***HR*** | ***95% CI*** | ***P value*** |
| TOFA mono | *Reference group* |  |  |  |  |  |
| TOFA + csDMARD | 0.83 | 0.68 1.03 | 0.09 | 1.15 | 0.92 1.44 | 0.22 |
| **ADA mono** | **0.74** | **0.63 0.87** | **<0.001** | 0.88 | 0.73 1.05 | 0.16 |
| **ADA + csDMARD** | **0.59** | **0.501 0.69** | **<0.001** | **0.74** | **0.61 0.89** | **0.002** |
| ^a^Drug discontinuation is defined by stop date of tofacitinib/adalimumab  *Adjusted for confounders: age at start TOFA/ADA, sex, ACPA +, smoking status, baseline DAS28, follow up time, previous b/tsDMARD use  ADA mono, adalimumab monotherapy; 95% CI, 95% confidence interval; csDMARD, conventional synthetic disease-modifying antirheumatic drug; DAS28, disease activity score assessed using 28 joints; HR, hazard ratio; TOFA, tofacitinib. | | | | | | |

## Table S1.19 Results of Cox regression model with the time to drug discontinuation as dependent variable and treatment groups as independent variable. Including an interaction term between treatment group and ACPA status^a^. Only including patients who started Adalimumab>2009. Drug discontinuation is defined by DAS28≥2.6 (no remission)^b^

|  | **Drug survival** | | | | |  |
| --- | --- | --- | --- | --- | --- | --- |
|  |  | **Unadjusted** |  |  | **Adjusted*** |  |
|  | ***HR*** | ***95% CI*** | ***P value*** | ***HR*** | ***95% CI*** | ***P value*** |
| TOFA mono #ACPA | *Reference group* |  |  |  |  |  |
| TOFA + csDMARD #ACPA | 0.94 | 0.54 1.65 | 0.82 | 0.86 | 0.49 1.47 | 0.58 |
| ADA mono #ACPA | 0.88 | 0.69 1.29 | 0.51 | 0.88 | 0.59 1.32 | 0.54 |
| ADA + csDMARD #ACPA | 0.75 | 0.51 1.09 | 0.14 | 0.78 | 0.52 1.17 | 0.23 |
| ^a^ACPA - is the reference  ^b^Drug discontinuation is defined by stop date of tofacitinib/adalimumab in combination with DAS≥2.6 (no remission)  *Adjusted for confounders: age at start TOFA/ADA, sex, smoking status, baseline DAS28, follow up time, previous b/tsDMARD use  ADA mono, adalimumab monotherapy; 95% CI, 95% confidence interval; csDMARD, conventional synthetic disease-modifying antirheumatic drug; DAS28, disease activity score assessed using 28 joints; HR, hazard ratio; TOFA, tofacitinib. | | | | | | |

## Table S1.20 Results of Cox regression model with the time to drug discontinuation^a^ as dependent variable and treatment groups as independent variable. Including an interaction term between treatment group and ACPA status^b^. Only including patients who started Adalimumab>2009

|  | **Drug survival** | | | | |  |
| --- | --- | --- | --- | --- | --- | --- |
|  |  | **Unadjusted** |  |  | **Adjusted*** |  |
|  | ***HR*** | ***95% CI*** | ***P value*** | ***HR*** | ***95% CI*** | ***P value*** |
| TOFA mono #ACPA | *Reference group* |  |  |  |  |  |
| TOFA + csDMARD #ACPA | 0.90 | 0.53 1.54 | 0.70 | 0.80 | 0.49 1.31 | 0.38 |
| ADA mono #ACPA | 0.97 | 0.68 1.377 | 0.85 | 0.94 | 0.65 1.34 | 0.72 |
| ADA + csDMARD #ACPA | 0.83 | 0.59 1.18 | 0.29 | 0.82 | 0.58 1.18 | 0.29 |
| ^a^Drug discontinuation is defined by stop date of tofacitinib/adalimumab  ^b^ACPA - is the reference  *Adjusted for confounders: age at start TOFA/ADA, sex, smoking status, baseline DAS28, previous b/tsDMARD use  ADA mono, adalimumab monotherapy; 95% CI, 95% confidence interval; csDMARD, conventional synthetic disease-modifying antirheumatic drug; DAS28, disease activity score assessed using 28 joints; HR, hazard ratio; TOFA, tofacitinib. | | | | | | |

## Table S1.21 Results of Cox regression model with the time to drug discontinuation as dependent variable and treatment groups as independent variable. Including an interaction term between treatment group and age^a^. Only including patients who started Adalimumab>2009. Drug discontinuation is defined by DAS28≥2.6 (no remission)^b^

|  | **Drug survival** | | | | |  |
| --- | --- | --- | --- | --- | --- | --- |
|  |  | **Unadjusted** |  |  | **Adjusted*** |  |
|  | ***HR*** | ***95% CI*** | ***P value*** | ***HR*** | ***95% CI*** | ***P value*** |
| TOFA mono #age | *Reference group* |  |  |  |  |  |
| **TOFA + csDMARD #age** | **1.01** | **0.99 1.03** | **0.09** | **1.02** | **0.99 1.04** | **0.07** |
| **ADA mono #age** | **1.01** | **0.99 1.03** | **0.07** | 1.01 | 0.99 1.03 | 0.11 |
| **ADA + csDMARD #age** | **1.02** | **1.01 1.03** | **0.003** | **1.02** | **1.01 1.03** | **0.01** |
| ^a^Age is continuous  ^b^Drug discontinuation is defined by stop date of tofacitinib/adalimumab in combination with DAS≥2.6 (no remission)  *Adjusted for confounders: sex, ACPA +, smoking status, baseline DAS28, follow up time, previous b/tsDMARD use  ADA mono, adalimumab monotherapy; 95% CI, 95% confidence interval; csDMARD, conventional synthetic disease-modifying antirheumatic drug; DAS28, disease activity score assessed using 28 joints; HR, hazard ratio; TOFA, tofacitinib | | | | | | |

## Table S1.22 Results of Cox regression model with the time to drug discontinuation^a^ as dependent variable and treatment groups as independent variable. Including an interaction term between treatment group and age^b^. Only including patients who started Adalimumab>2009

|  | **Drug survival** | | | | |  |
| --- | --- | --- | --- | --- | --- | --- |
|  |  | **Unadjusted** |  |  | **Adjusted*** |  |
|  | ***HR*** | ***95% CI*** | ***P value*** | ***HR*** | ***95% CI*** | ***P value*** |
| TOFA mono #age | *Reference group* |  |  |  |  |  |
| TOFA + csDMARD #age | 1.01 | 0.99 1.03 | 0.11 | 1.01 | 0.99 1.03 | 0.12 |
| ADA mono #age | 1.01 | 0.99 1.02 | 0.37 | 1.01 | 0.99 1.02 | 0.29 |
| **ADA + csDMARD #age** | **1.01** | **0.99 1.03** | **0.06** | **1.01** | **0.99 1.03** | **0.08** |
| ^a^Drug discontinuation is defined by stop date of tofacitinib/adalimumab  ^b^Age is continuous  *Adjusted for confounders: sex, ACPA +, smoking status, baseline DAS28, follow up time, previous b/tsDMARD use  ADA mono, adalimumab monotherapy; 95% CI, 95% confidence interval; csDMARD, conventional synthetic disease-modifying antirheumatic drug; DAS28, disease activity score assessed using 28 joints; HR, hazard ratio; TOFA, tofacitinib. | | | | | | |

## Table S1.23 Results of Cox regression model with the time to drug discontinuation as dependent variable and treatment groups as independent variable. Only including patients who started Adalimumab>2012. Drug discontinuation is defined by DAS28≥2.6 (no remission)^a^

|  | **Drug survival** | | | | |  |
| --- | --- | --- | --- | --- | --- | --- |
|  |  | **Unadjusted** |  |  | **Adjusted*** |  |
|  | ***HR*** | ***95% CI*** | ***P value*** | ***HR*** | ***95% CI*** | ***P value*** |
| TOFA mono | *Reference group* |  |  |  |  |  |
| TOFA + csDMARD | 0.89 | 0.71 1.10 | 0.28 | 1.19 | 0.94 1.52 | 0.14 |
| **ADA mono** | **0.76** | **0.64 0.90** | **0.002** | 0.87 | 0.71 1.07 | 0.18 |
| **ADA + csDMARD** | **0.65** | **0.55 0.77** | **<0.001** | **0.74** | **0.59 0.91** | **0.01** |
| ^a^Drug discontinuation is defined by stop date of tofacitinib/adalimumab in combination with DAS≥2.6 (no remission)  *Adjusted for confounders: age at start TOFA/ADA, sex, ACPA +, smoking status, baseline DAS28, follow up time, previous b/tsDMARD use  ADA mono, adalimumab monotherapy; 95% CI, 95% confidence interval; csDMARD, conventional synthetic disease-modifying antirheumatic drug; DAS28, disease activity score assessed using 28 joints; HR, hazard ratio; TOFA, tofacitinib. | | | | | | |

## Table S1.24 Results of Cox regression model with the time to drug discontinuation^a^ as dependent variable and treatment groups as independent variable. Only including patients who started Adalimumab>2012

|  | **Drug survival** | | | | |  |
| --- | --- | --- | --- | --- | --- | --- |
|  |  | **Unadjusted** |  |  | **Adjusted*** |  |
|  | ***HR*** | ***95% CI*** | ***P value*** | ***HR*** | ***95% CI*** | ***P value*** |
| TOFA mono | *Reference group* |  |  |  |  |  |
| TOFA + csDMARD | 0.83 | 0.67 1.03 | 0.09 | 1.14 | 0.91 1.42 | 0.26 |
| **ADA mono** | **0.73** | **0.62 0.86** | **<0.001** | 0.85 | 0.71 1.03 | 0.09 |
| **ADA + csDMARD** | **0.64** | **0.55 0.75** | **<0.001** | **0.73** | **0.60 0.89** | **0.002** |
| ^a^Drug discontinuation is defined by stop date of tofacitinib/adalimumab  *Adjusted for confounders: age at start TOFA/ADA, sex, ACPA +, smoking status, baseline DAS28, follow up time, previous b/tsDMARD use  ADA mono, adalimumab monotherapy; 95% CI, 95% confidence interval; csDMARD, conventional synthetic disease-modifying antirheumatic drug; DAS28, disease activity score assessed using 28 joints; HR, hazard ratio; TOFA, tofacitinib. | | | | | | |

## Table S1.25 Results of Cox regression model with the time to drug discontinuation as dependent variable and treatment groups as independent variable. Including an interaction term between treatment group and ACPA status^a^. Only including patients who started Adalimumab>2012. Drug discontinuation is defined by DAS28≥2.6 (no remission)^b^

|  | **Drug survival** | | | | |  |
| --- | --- | --- | --- | --- | --- | --- |
|  |  | **Unadjusted** |  |  | **Adjusted*** |  |
|  | ***HR*** | ***95% CI*** | ***P value*** | ***HR*** | ***95% CI*** | ***P value*** |
| TOFA mono #ACPA | *Reference group* |  |  |  |  |  |
| TOFA + csDMARD #ACPA | 0.94 | 0.53 1.65 | 0.82 | 0.86 | 0.50 1.46 | 0.57 |
| ADA mono #ACPA | 0.87 | 0.59 1.29 | 0.49 | 0.87 | 0.58 1.30 | 0.49 |
| ADA + csDMARD #ACPA | 0.79 | 0.54 1.17 | 0.24 | 0.80 | 0.53 1.21 | 0.29 |
| ^a^ACPA - is the reference  ^b^Drug discontinuation is defined by stop date of tofacitinib/adalimumab in combination with DAS≥2.6 (no remission)  *Adjusted for confounders: age at start TOFA/ADA, sex, smoking status, baseline DAS28, follow up time, previous b/tsDMARD use  ADA mono, adalimumab monotherapy; 95% CI, 95% confidence interval; csDMARD, conventional synthetic disease-modifying antirheumatic drug; DAS28, disease activity score assessed using 28 joints; HR, hazard ratio; TOFA, tofacitinib. | | | | | | |

## Table S1.26 Results of Cox regression model with the time to drug discontinuation^a^ as dependent variable and treatment groups as independent variable. Including an interaction term between treatment group and ACPA status^b^. Only including patients who started Adalimumab>2012

|  | **Drug survival** | | | | |  |
| --- | --- | --- | --- | --- | --- | --- |
|  |  | **Unadjusted** |  |  | **Adjusted*** |  |
|  | ***HR*** | ***95% CI*** | ***P value*** | ***HR*** | ***95% CI*** | ***P value*** |
| TOFA mono #ACPA | *Reference group* |  |  |  |  |  |
| TOFA + csDMARD #ACPA | 0.89 | 0.52 1.544 | 0.69 | 0.79 | 0.49 1.30 | 0.37 |
| ADA mono #ACPA | 0.94 | 0.66 1.35 | 0.75 | 0.92 | 0.64 1.32 | 0.64 |
| ADA + csDMARD #ACPA | 0.87 | 0.61 1.24 | 0.44 | 0.84 | 0.59 1.21 | 0.35 |
| ^a^Drug discontinuation is defined by stop date of tofacitinib/adalimumab  ^b^ACPA - is the reference  *Adjusted for confounders: age at start TOFA/ADA, sex, smoking status, baseline DAS28, previous b/tsDMARD use  ADA mono, adalimumab monotherapy; 95% CI, 95% confidence interval; csDMARD, conventional synthetic disease-modifying antirheumatic drug; DAS28, disease activity score assessed using 28 joints; HR, hazard ratio; TOFA, tofacitinib. | | | | | | |

## Table S1.27 Results of Cox regression model with the time to drug discontinuation as dependent variable and treatment groups as independent variable. Including an interaction term between treatment group and age^a^. Only including patients who started Adalimumab>2012. Drug discontinuation is defined by DAS28≥2.6 (no remission)^b^

|  | **Drug survival** | | | | |  |
| --- | --- | --- | --- | --- | --- | --- |
|  |  | **Unadjusted** |  |  | **Adjusted*** |  |
|  | ***HR*** | ***95% CI*** | ***P value*** | ***HR*** | ***95% CI*** | ***P value*** |
| TOFA mono #age | *Reference group* |  |  |  |  |  |
| **TOFA + csDMARD #age** | **1.01** | **0.99 1.03** | **0.09** | **1.02** | **0.99 1.04** | **0.07** |
| **ADA mono #age** | **1.01** | **0.99 1.03** | **0.09** | 1.01 | 0.99 1.03 | 0.11 |
| **ADA + csDMARD #age** | **1.02** | **1.01 1.03** | **0.01** | **1.02** | **1.004 1.03** | **0.01** |
| ^a^Age is continuous  ^b^Drug discontinuation is defined by stop date of tofacitinib/adalimumab in combination with DAS≥2.6 (no remission)  *Adjusted for confounders: sex, ACPA +, smoking status, baseline DAS28, follow up time, previous b/tsDMARD use  ADA mono, adalimumab monotherapy; 95% CI, 95% confidence interval; csDMARD, conventional synthetic disease-modifying antirheumatic drug; DAS28, disease activity score assessed using 28 joints; HR, hazard ratio; TOFA, tofacitinib | | | | | | |

## Table S1.28 Results of Cox regression model with the time to drug discontinuation^a^ as dependent variable and treatment groups as independent variable. Including an interaction term between treatment group and age^b^. Only including patients who started Adalimumab>2012

|  | **Drug survival** | | | | |  |
| --- | --- | --- | --- | --- | --- | --- |
|  |  | **Unadjusted** |  |  | **Adjusted*** |  |
|  | ***HR*** | ***95% CI*** | ***P value*** | ***HR*** | ***95% CI*** | ***P value*** |
| TOFA mono #age | *Reference group* |  |  |  |  |  |
| TOFA + csDMARD #age | 1.01 | 0.99 1.03 | 0.11 | 1.02 | 0.99 1.03 | 0.11 |
| ADA mono #age | 1.01 | 0.99 1.02 | 0.37 | 1.01 | 0.99 1.02 | 0.23 |
| **ADA + csDMARD #age** | **1.01** | **0.99 1.02** | **0.09** | **1.01** | **0.99 1.03** | **0.09** |
| ^a^Drug discontinuation is defined by stop date of tofacitinib/adalimumab  ^b^Age is continuous  *Adjusted for confounders: sex, ACPA +, smoking status, baseline DAS28, follow up time, previous b/tsDMARD use  ADA mono, adalimumab monotherapy; 95% CI, 95% confidence interval; csDMARD, conventional synthetic disease-modifying antirheumatic drug; DAS28, disease activity score assessed using 28 joints; HR, hazard ratio; TOFA, tofacitinib. | | | | | | |

## Table S1.29 Results of Cox regression model with the time to drug discontinuation as dependent variable and treatment groups as independent variable. Only including patients who started Tofacitinib/Adalimumab with no previous b/tsDMARD use. Drug discontinuation is defined by DAS28≥2.6 (no remission)^a^

|  | **Drug survival** | | | | |  |
| --- | --- | --- | --- | --- | --- | --- |
|  |  | **Unadjusted** |  |  | **Adjusted*** |  |
|  | ***HR*** | ***95% CI*** | ***P value*** | ***HR*** | ***95% CI*** | ***P value*** |
| TOFA mono | *Reference group* |  |  |  |  |  |
| TOFA + csDMARD | 0.78 | 0.55 1.09 | 0.14 | 0.99 | 0.69 1.45 | 0.99 |
| **ADA mono** | **0.59** | **0.46 0.78** | **<0.001** | **0.70** | **0.52 0.95** | **0.02** |
| **ADA + csDMARD** | **0.54** | **0.41 0.69** | **<0.001** | **0.61** | **0.45 0.83** | **0.001** |
| ^a^Drug discontinuation is defined by stop date of tofacitinib/adalimumab in combination with DAS≥2.6 (no remission)  *Adjusted for confounders: age at start TOFA/ADA, sex, ACPA +, smoking status, baseline DAS28, follow up time  ADA mono, adalimumab monotherapy; 95% CI, 95% confidence interval; csDMARD, conventional synthetic disease-modifying antirheumatic drug; DAS28, disease activity score assessed using 28 joints; HR, hazard ratio; TOFA, tofacitinib | | | | | | |

## Table S1.30 Results of Cox regression model with the time to drug discontinuation^a^ as dependent variable and treatment groups as independent variable. Only including patients who started Tofacitinib/Adalimumab with no previous b/tsDMARD use

|  | **Drug survival** | | | | |  |
| --- | --- | --- | --- | --- | --- | --- |
|  |  | **Unadjusted** |  |  | **Adjusted*** |  |
|  | ***HR*** | ***95% CI*** | ***P value*** | ***HR*** | ***95% CI*** | ***P value*** |
| TOFA mono | *Reference group* |  |  |  |  |  |
| TOFA + csDMARD | 0.73 | 0.53 1.00 | 0.05 | 0.99 | 0.69 1.39 | 0.95 |
| **ADA mono** | **0.70** | **0.55 0.89** | **0.003** | **0.68** | **0.52 0.89** | **0.01** |
| **ADA + csDMARD** | **0.56** | **0.45 0.71** | **<0.001** | **0.61** | **0.47 0.802** | **<0.001** |
| ^a^Drug discontinuation is defined by stop date of tofacitinib/adalimumab  *Adjusted for confounders: age at start TOFA/ADA, sex, ACPA +, smoking status, baseline DAS28, follow up time  ADA mono, adalimumab monotherapy; 95% CI, 95% confidence interval; csDMARD, conventional synthetic disease-modifying antirheumatic drug; DAS28, disease activity score assessed using 28 joints; HR, hazard ratio; TOFA, tofacitinib | | | | | | |

## Table S1.31 Baseline characteristics per treatment group: only including patients with no prior b/tsDMARD use

|  | **TOFA monotherapy** | **TOFA + csDMARD** | *P value* | **ADA monotherapy** | *P value* | **ADA + csDMARD** | *P value* |
| --- | --- | --- | --- | --- | --- | --- | --- |
|  | No previous b/tsDMARD | No previous b/tsDMARD |  | No previous b/tsDMARD |  | No previous b/tsDMARD |  |
| N, % of total | 58 (3) | 49 (2) |  | 819 (39) |  | 1191 (56) |  |
| Age at start biological^a^, mean (SD) | 60 (13) | 53 (15) | **0.01** | 57 (14) | *0.074* | 58 (13) | *0.11* |
| *Missingness, %* | *0* | *0* |  | *0* |  | *0.4* |  |
| Year that biological^a^ was started, median (IQR) | 2020  (2019-2021) | 2019  (2019-2019) | **<0.001** | 2017  (2014-2019) | **<0.001** | 2017  (2014-2019) | **<0.001** |
| Female, % | 74 | 78 | 0.68 | 69 | 0.41 | 67 | 0.26 |
| *Missingness, %* | *0* | *0* |  | *0.1* |  | *0.3* |  |
| RF+, % | 33 | 35 | 0.09 | 44 | 0.26 | 53 | 0.06 |
| *Missingness, %* | *48* | *59* |  | *40* |  | *32* |  |
| ACPA, % | 64 | 35 | 0.88 | 40 | 0.30 | 49 | 0.98 |
| *Missingness, %* | *12* | *51* |  | *39* |  | *33* |  |
| Smoker, % | 28 | 45 | 0.46 | 29 | **0.04** | 30 | 0.07 |
| *Missingness, %* | *60* | *31* |  | *53* |  | *49* |  |
| DAS28, mean (SD) | 3.1 (1.4) | 3.5 (1.2) | 0.35 | 3.5 (1.5) | 0.16 | 3.6 (1.5) | 0.11 |
| *Missingness, %* | *62* | *65* |  | *48* |  | *50* |  |
| ^a^Tofacitinib or Adalimumab  ADA mono, adalimumab monotherapy; 95% CI, 95% confidence interval; b/tsDMARDs, biologic/targeted synthetic disease-modifying antirheumatic drugs; csDMARD, conventional synthetic disease-modifying antirheumatic drug; DAS28, disease activity score assessed using 28 joints; RF+, Rheumatoid Factor positive; Smoker, patients who were smokers or who had ever smoked; TOFA, tofacitinib  ^b^ *P values reflect comparisons with TOFA monotherapy as reference group* | | | | | | | |
