# Supplementary material for: Real-world effectiveness of tofacitinib in monotherapy or in combination with csDMARDs in patients with rheumatoid arthritis: A collaboration between Dutch registries (TOFADUDAP study)
Source: Clin Rheumatol. 2026 Aug 19;45(10):6071–80. doi: 10.1007/s10067-026-08362-x (PMC13582166; doi:10.1007/s10067-026-08362-x)
Supplement: Supplementary file 2 [file 10067_2026_8362_MOESM2_ESM.docx]

**SUPPLEMENTARY INFORMATION 2**

Analyses on multiple imputed data including the assumption that previous b/tsDMARD use is 0 if previous b/tsDMARD use is missing and tofacitinib is initiated at first visit.

**DRUG SURVIVAL**

Table S2.1 Results of Cox regression model with time to drug discontinuation as dependent variable and treatment group as independent variable. Drug discontinuation is defined by DAS28≥2.6 (no remission)^a^

|  | **Drug survival** | | | | |  |
| --- | --- | --- | --- | --- | --- | --- |
|  |  | **Unadjusted** |  |  | **Adjusted*** |  |
|  | ***HR*** | ***95% CI*** | ***P value*** | ***HR*** | ***95% CI*** | ***P value*** |
| TOFA mono | *Reference group* |  |  |  |  |  |
| TOFA + csDMARD | 0.89 | 0.72 1.11 | 0.29 | 1.20 | 0.95 1.52 | 0.13 |
| **ADA mono** | **0.69** | **0.58 0.82** | **<0.001** | 0.83 | 0.68 1.02 | 0.07 |
| **ADA + csDMARD** | **0.57** | **0.48 0.67** | **<0.001** | **0.72** | **0.59 0.89** | **0.002** |
| ^a^Drug discontinuation is defined by stop date of tofacitinib/adalimumab in combination with DAS≥2.6 (no remission)  *Adjusted for confounders: age at start TOFA/ADA, sex, ACPA +, smoking status, baseline DAS28, follow up time, previous b/tsDMARD use  ADA mono, adalimumab monotherapy; 95% CI, 95% confidence interval; csDMARD, conventional synthetic disease-modifying antirheumatic drug; DAS28, disease activity score assessed using 28 joints; HR, hazard ratio; TOFA, tofacitinib. | | | | | | |

Table S2.2 Results of Cox regression model with time to drug discontinuation^a^ as dependent variable and treatment group as independent variable

|  | **Drug survival** | | | | |  |
| --- | --- | --- | --- | --- | --- | --- |
|  |  | **Unadjusted** |  |  | **Adjusted*** |  |
|  | ***HR*** | ***95% CI*** | ***P value*** | ***HR*** | ***95% CI*** | ***P value*** |
| TOFA mono | *Reference group* |  |  |  |  |  |
| TOFA + csDMARD | 0.84 | 0.68 1.03 | 0.09 | 1.14 | 0.91 1.42 | 0.25 |
| **ADA mono** | **0.69** | **0.59 0.80** | **<0.001** | **0.81** | **0.68 0.97** | **0.02** |
| **ADA + csDMARD** | **0.57** | **0.48 0.66** | **<0.001** | **0.72** | **0.59 0.86** | **<0.001** |
| ^a^Drug discontinuation is defined by stop date of tofacitinib/adalimumab  *Adjusted for confounders: age at start TOFA/ADA, sex, ACPA +, smoking status, baseline DAS28, follow up time, previous b/tsDMARD use  ADA mono, adalimumab monotherapy; 95% CI, 95% confidence interval; csDMARD, conventional synthetic disease-modifying antirheumatic drug; DAS28, disease activity score assessed using 28 joints; HR, hazard ratio; TOFA, tofacitinib. | | | | | | |

Table S2.3 Results of Cox regression model with time to drug discontinuation as dependent variable and treatment group as independent variable. Including an interaction term between treatment group and ACPA status^a^. Drug discontinuation is defined by DAS28≥2.6 (no remission)^b^

|  | **Drug survival** | | | | |  |
| --- | --- | --- | --- | --- | --- | --- |
|  |  | **Unadjusted** |  |  | **Adjusted*** |  |
|  | ***HR*** | ***95% CI*** | ***P value*** | ***HR*** | ***95% CI*** | ***P value*** |
| TOFA mono #ACPA | *Reference group* |  |  |  |  |  |
| TOFA + csDMARD #ACPA | 0.99 | 0.55 1.79 | 0.98 | 0.93 | 0.54 1.62 | 0.80 |
| ADA mono #ACPA | 0.88 | 0.60 1.28 | 0.49 | 0.92 | 0.61 1.38 | 0.68 |
| ADA + csDMARD #ACPA | 0.74 | 0.51 1.08 | 0.12 | 0.82 | 0.55 1.21 | 0.31 |
| ^a^ACPA - is the reference  ^b^Drug discontinuation is defined by stop date of tofacitinib/adalimumab in combination with DAS≥2.6 (no remission)  *Adjusted for confounders: age at start TOFA/ADA, sex, smoking status, baseline DAS28, follow up time, previous b/tsDMARD use  ADA mono, adalimumab monotherapy; 95% CI, 95% confidence interval; csDMARD, conventional synthetic disease-modifying antirheumatic drug; DAS28, disease activity score assessed using 28 joints; HR, hazard ratio; TOFA, tofacitinib. | | | | | | |

Table S2.4 Results of Cox regression model with time to drug discontinuation^a^ as dependent variable and treatment group as independent variable. Including an interaction term between treatment group and ACPA status^b^

|  | **Drug survival** | | | | |  |
| --- | --- | --- | --- | --- | --- | --- |
|  |  | **Unadjusted** |  |  | **Adjusted*** |  |
|  | ***HR*** | ***95% CI*** | ***P value*** | ***HR*** | ***95% CI*** | ***P value*** |
| TOFA mono #ACPA | *Reference group* |  |  |  |  |  |
| TOFA + csDMARD #ACPA | 0.95 | 0.54 1.68 | 0.87 | 0.88 | 0.53 1.44 | 0.61 |
| ADA mono #ACPA | 0.96 | 0.68 1.35 | 0.80 | 0.97 | 0.68 1.39 | 0.87 |
| ADA + csDMARD #ACPA | 0.83 | 0.58 1.18 | 0.29 | 0.87 | 0.61 1.24 | 0.45 |
| ^a^Drug discontinuation is defined by stop date of tofacitinib/adalimumab  ^b^ACPA - is the reference  *Adjusted for confounders: age at start TOFA/ADA, sex, smoking status, baseline DAS28, follow up time, previous b/tsDMARD use  ADA mono, adalimumab monotherapy; 95% CI, 95% confidence interval; csDMARD, conventional synthetic disease-modifying antirheumatic drug; DAS28, disease activity score assessed using 28 joints; HR, hazard ratio; TOFA, tofacitinib. | | | | | | |

Table S2.5 Results of Cox regression model with time to drug discontinuation as dependent variable and treatment group as independent variable. Including an interaction term between treatment group and age^a^. Drug discontinuation is defined by DAS28≥2.6 (no remission)^b^

|  | **Drug survival** | | | | |  |
| --- | --- | --- | --- | --- | --- | --- |
|  |  | **Unadjusted** |  |  | **Adjusted*** |  |
|  | ***HR*** | ***95% CI*** | ***P value*** | ***HR*** | ***95% CI*** | ***P value*** |
| TOFA mono #age | *Reference group* |  |  |  |  |  |
| **TOFA + csDMARD #age** | **1.01** | **0.99 1.03** | **0.09** | **1.02** | **0.99 1.04** | **0.05** |
| **ADA mono #age** | **1.02** | **1.004 1.03** | **0.01** | **1.02** | **1.00 1.03** | **0.05** |
| **ADA + csDMARD #age** | **1.02** | **1.01 1.03** | **0.01** | **1.02** | **1.00 1.03** | **0.02** |
| ^a^Age is continuous  ^b^Drug discontinuation is defined by stop date of tofacitinib/adalimumab in combination with DAS≥2.6 (no remission)  *Adjusted for confounders: sex, ACPA +, smoking status, baseline DAS28, follow up time, previous b/tsDMARD use  ADA mono, adalimumab monotherapy; 95% CI, 95% confidence interval; csDMARD, conventional synthetic disease-modifying antirheumatic drug; DAS28, disease activity score assessed using 28 joints; HR, hazard ratio; TOFA, tofacitinib. | | | | | | |

Table S2.6 Results of Cox regression model with time to drug discontinuation^a^ as dependent variable and treatment group as independent variable. Including an interaction term between treatment group and age^b^

|  | **Drug survival** | | | | |  |
| --- | --- | --- | --- | --- | --- | --- |
|  |  | **Unadjusted** |  |  | **Adjusted*** |  |
|  | ***HR*** | ***95% CI*** | ***P value*** | ***HR*** | ***95% CI*** | ***P value*** |
| **TOFA mono #age** | *Reference group* |  |  |  |  |  |
| **TOFA + csDMARD #age** | 1.01 | 0.99 1.03 | 0.11 | **1.02** | **0.99 1.03** | **0.09** |
| ADA mono #age | 1.01 | 0.99 1.02 | 0.13 | 1.01 | 0.99 1.02 | 0.15 |
| **ADA + csDMARD #age** | **1.01** | **0.99 1.02** | **0.09** | 1.01 | 0.99 1.02 | 0.12 |
| ^a^Drug discontinuation is defined by stop date of tofacitinib/adalimumab  ^b^Age is continuous  *Adjusted for confounders: sex, ACPA +, smoking status, baseline DAS28, follow up time, previous b/tsDMARD use  ADA mono, adalimumab monotherapy; 95% CI, 95% confidence interval; csDMARD, conventional synthetic disease-modifying antirheumatic drug; DAS28, disease activity score assessed using 28 joints; HR, hazard ratio; TOFA, tofacitinib. | | | | | | |

**SUPPLEMENTARY MATERIAL 3**

Analyses on multiple imputed data including the assumption that previous b/tsDMARD use is 1 if previous b/tsDMARD use is missing and tofacitinib is initiated at first visit.

**DRUG SURVIVAL**

Table S3.1 Results of Cox regression model with time to drug discontinuation as dependent variable and treatment group as independent variable. Drug discontinuation is defined by DAS28≥2.6 (no remission)^a^

|  | **Drug survival** | | | | |  |
| --- | --- | --- | --- | --- | --- | --- |
|  |  | **Unadjusted** |  |  | **Adjusted*** |  |
|  | ***HR*** | ***95% CI*** | ***P value*** | ***HR*** | ***95% CI*** | ***P value*** |
| TOFA mono | *Reference group* |  |  |  |  |  |
| TOFA + csDMARD | 0.89 | 0.72 1.11 | 0.29 | 1.21 | 0.96 1.53 | 0.11 |
| **ADA mono** | **0.69** | **0.58 0.82** | **<0.001** | 0.99 | 0.80 1.23 | 0.93 |
| **ADA + csDMARD** | **0.57** | **0.48 0.67** | **<0.001** | 0.87 | 0.69 1.08 | 0.19 |
| ^a^Drug discontinuation is defined by stop date of tofacitinib/adalimumab in combination with DAS≥2.6 (no remission)  *Adjusted for confounders: age at start TOFA/ADA, sex, ACPA +, smoking status, baseline DAS28, follow up time, previous b/tsDMARD use  ADA mono, adalimumab monotherapy; 95% CI, 95% confidence interval; csDMARD, conventional synthetic disease-modifying antirheumatic drug; DAS28, disease activity score assessed using 28 joints; HR, hazard ratio; TOFA, tofacitinib. | | | | | | |

Table S3.2 Results of Cox regression model with time to drug discontinuation^a^ as dependent variable and treatment group as independent variable

|  | **Drug survival** | | | | |  |
| --- | --- | --- | --- | --- | --- | --- |
|  |  | **Unadjusted** |  |  | **Adjusted*** |  |
|  | ***HR*** | ***95% CI*** | ***P value*** | ***HR*** | ***95% CI*** | ***P value*** |
| TOFA mono | *Reference group* |  |  |  |  |  |
| TOFA + csDMARD | 0.84 | 0.68 1.03 | 0.09 | 1.14 | 0.92 1.42 | 0.24 |
| **ADA mono** | **0.69** | **0.59 0.80** | **<0.001** | 0.96 | 0.79 1.16 | 0.65 |
| **ADA + csDMARD** | **0.57** | **0.48 0.66** | **<0.001** | 0.85 | 0.69 1.04 | 0.11 |
| ^a^Drug discontinuation is defined by stop date of tofacitinib/adalimumab  *Adjusted for confounders: age at start TOFA/ADA, sex, ACPA +, smoking status, baseline DAS28, follow up time, previous b/tsDMARD use  ADA mono, adalimumab monotherapy; 95% CI, 95% confidence interval; csDMARD, conventional synthetic disease-modifying antirheumatic drug; DAS28, disease activity score assessed using 28 joints; HR, hazard ratio; TOFA, tofacitinib. | | | | | | |

Table S3.3 Results of Cox regression model with time to drug discontinuation as dependent variable and treatment group as independent variable. Including an interaction term between treatment group and ACPA status^a^. Drug discontinuation is defined by DAS28≥2.6 (no remission)^b^

|  | **Drug survival** | | | | |  |
| --- | --- | --- | --- | --- | --- | --- |
|  |  | **Unadjusted** |  |  | **Adjusted*** |  |
|  | ***HR*** | ***95% CI*** | ***P value*** | ***HR*** | ***95% CI*** | ***P value*** |
| TOFA mono #ACPA | *Reference group* |  |  |  |  |  |
| TOFA + csDMARD #ACPA | 0.95 | 0.54 1.66 | 0.86 | 0.82 | 0.48 1.39 | 0.46 |
| ADA mono #ACPA | 0.87 | 0.58 1.31 | 0.51 | 0.84 | 0.56 1.27 | 0.41 |
| ADA + csDMARD #ACPA | 0.73 | 0.49 1.09 | 0.12 | 0.74 | 0.49 1.09 | 0.13 |
| ^a^ACPA - is the reference  ^b^Drug discontinuation is defined by stop date of tofacitinib/adalimumab in combination with DAS≥2.6 (no remission)  *Adjusted for confounders: age at start TOFA/ADA, sex, smoking status, baseline DAS28, follow up time, previous b/tsDMARD use  ADA mono, adalimumab monotherapy; 95% CI, 95% confidence interval; csDMARD, conventional synthetic disease-modifying antirheumatic drug; DAS28, disease activity score assessed using 28 joints; HR, hazard ratio; TOFA, tofacitinib. | | | | | | |

Table S3.4 Results of Cox regression model with time to drug discontinuation^a^ as dependent variable and treatment group as independent variable. Including an interaction term between treatment group and ACPA status^b^

|  | **Drug survival** | | | | |  |
| --- | --- | --- | --- | --- | --- | --- |
|  |  | **Unadjusted** |  |  | **Adjusted*** |  |
|  | ***HR*** | ***95% CI*** | ***P value*** | ***HR*** | ***95% CI*** | ***P value*** |
| TOFA mono #ACPA | *Reference group* |  |  |  |  |  |
| TOFA + csDMARD #ACPA | 0.92 | 0.54 1.57 | 0.75 | 0.78 | 0.48 1.26 | 0.31 |
| ADA mono #ACPA | 0.95 | 0.65 1.39 | 0.79 | 0.89 | 0.62 1.29 | 0.56 |
| ADA + csDMARD #ACPA | 0.82 | 0.57 1.19 | 0.29 | 0.79 | 0.56 1.14 | 0.21 |
| ^b^ACPA - is the reference  ^b^Drug discontinuation is defined by stop date of tofacitinib/adalimumab  *Adjusted for confounders: age at start TOFA/ADA, sex, smoking status, baseline DAS28, follow up time, previous b/tsDMARD use  ADA mono, adalimumab monotherapy; 95% CI, 95% confidence interval; csDMARD, conventional synthetic disease-modifying antirheumatic drug; DAS28, disease activity score assessed using 28 joints; HR, hazard ratio; TOFA, tofacitinib. | | | | | | |

Table S3.5 Results of Cox regression model with time to drug discontinuation as dependent variable and treatment group as independent variable. Including an interaction term between treatment group and age^a^. Drug discontinuation is defined by DAS28≥2.6 (no remission)^b^

|  | **Drug survival** | | | | |  |
| --- | --- | --- | --- | --- | --- | --- |
|  |  | **Unadjusted** |  |  | **Adjusted*** |  |
|  | ***HR*** | ***95% CI*** | ***P value*** | ***HR*** | ***95% CI*** | ***P value*** |
| TOFA mono #age | *Reference group* |  |  |  |  |  |
| **TOFA + csDMARD #age** | **1.01** | **0.99 1.03** | **0.09** | **1.02** | **0.99 1.03** | **0.08** |
| **ADA mono #age** | **1.02** | **1.003 1.03** | **0.01** | **1.02** | **1.001 1.03** | **0.04** |
| **ADA + csDMARD #age** | **1.02** | **1.01 1.03** | **0.01** | **1.02** | **1.004 1.03** | **0.01** |
| ^a^Age is continuous  ^b^Drug discontinuation is defined by stop date of tofacitinib/adalimumab in combination with DAS≥2.6 (no remission)  *Adjusted for confounders: sex, ACPA +, smoking status, baseline DAS28, follow up time, previous b/tsDMARD use  ADA mono, adalimumab monotherapy; 95% CI, 95% confidence interval; csDMARD, conventional synthetic disease-modifying antirheumatic drug; DAS28, disease activity score assessed using 28 joints; HR, hazard ratio; TOFA, tofacitinib. | | | | | | |

Table S3.6 Results of Cox regression model with time to drug discontinuation as dependent variable and treatment group as independent variable. Including an interaction term between treatment group and age^a^. Drug discontinuation is defined by DAS28≥2.6 (no remission)^b^

|  | **Drug survival** | | | | |  |
| --- | --- | --- | --- | --- | --- | --- |
|  |  | **Unadjusted** |  |  | **Adjusted*** |  |
|  | ***HR*** | ***95% CI*** | ***P value*** | ***HR*** | ***95% CI*** | ***P value*** |
| TOFA mono #age | *Reference group* |  |  |  |  |  |
| **TOFA + csDMARD #age** | **1.01** | **0.99 1.03** | **0.09** | **1.02** | **0.99 1.03** | **0.08** |
| **ADA mono #age** | **1.02** | **1.003 1.03** | **0.01** | **1.02** | **1.001 1.03** | **0.04** |
| **ADA + csDMARD #age** | **1.02** | **1.01 1.03** | **0.01** | **1.02** | **1.004 1.03** | **0.01** |
| ^a^Age is continuous  ^b^Drug discontinuation is defined by stop date of tofacitinib/adalimumab in combination with DAS≥2.6 (no remission)  *Adjusted for confounders: sex, ACPA +, smoking status, baseline DAS28, follow up time, previous b/tsDMARD use  ADA mono, adalimumab monotherapy; 95% CI, 95% confidence interval; csDMARD, conventional synthetic disease-modifying antirheumatic drug; DAS28, disease activity score assessed using 28 joints; HR, hazard ratio; TOFA, tofacitinib. | | | | | | |

**SUPPLEMENTARY MATERIAL 4**

Analyses on multiple imputed data including the assumption that previous b/tsDMARD use is 2 if previous b/tsDMARD use is missing and tofacitinib is initiated at first visit.

**DRUG SURVIVAL**

Table S4.1 Results of Cox regression model with time to drug discontinuation as dependent variable and treatment group as independent variable. Drug discontinuation is defined by DAS28≥2.6 (no remission)^a^

|  | **Drug survival** | | | | |  |
| --- | --- | --- | --- | --- | --- | --- |
|  |  | **Unadjusted** |  |  | **Adjusted*** |  |
|  | ***HR*** | ***95% CI*** | ***P value*** | ***HR*** | ***95% CI*** | ***P value*** |
| TOFA mono | *Reference group* |  |  |  |  |  |
| TOFA + csDMARD | 0.89 | 0.72 1.11 | 0.29 | 1.21 | 0.96 1.53 | 0.11 |
| **ADA mono** | **0.69** | **0.58 0.82** | **<0.001** | 0.99 | 0.78 1.27 | 0.95 |
| **ADA + csDMARD** | **0.57** | **0.48 0.67** | **<0.001** | 0.87 | 0.67 1.11 | 0.26 |
| ^a^Drug discontinuation is defined by stop date of tofacitinib/adalimumab in combination with DAS≥2.6 (no remission)  *Adjusted for confounders: age at start TOFA/ADA, sex, ACPA +, smoking status, baseline DAS28, follow up time, previous b/tsDMARD use  ADA mono, adalimumab monotherapy; 95% CI, 95% confidence interval; csDMARD, conventional synthetic disease-modifying antirheumatic drug; DAS28, disease activity score assessed using 28 joints; HR, hazard ratio; TOFA, tofacitinib. | | | | | | |

Table S4.2 Results of Cox regression model with time to drug discontinuation^a^ as dependent variable and treatment group as independent variable

|  | **Drug survival** | | | | |  |
| --- | --- | --- | --- | --- | --- | --- |
|  |  | **Unadjusted** |  |  | **Adjusted*** |  |
|  | ***HR*** | ***95% CI*** | ***P value*** | ***HR*** | ***95% CI*** | ***P value*** |
| TOFA mono | *Reference group* |  |  |  |  |  |
| TOFA + csDMARD | 0.84 | 0.68 1.03 | 0.09 | 1.14 | 0.92 1.42 | 0.24 |
| **ADA mono** | **0.69** | **0.59 0.80** | **<0.001** | 0.94 | 0.75 1.18 | 0.60 |
| **ADA + csDMARD** | **0.57** | **0.48 0.66** | **<0.001** | 0.83 | 0.66 1.05 | 0.13 |
| ^a^Drug discontinuation is defined by stop date of tofacitinib/adalimumab  *Adjusted for confounders: age at start TOFA/ADA, sex, ACPA +, smoking status, baseline DAS28, follow up time, previous b/tsDMARD use  ADA mono, adalimumab monotherapy; 95% CI, 95% confidence interval; csDMARD, conventional synthetic disease-modifying antirheumatic drug; DAS28, disease activity score assessed using 28 joints; HR, hazard ratio; TOFA, tofacitinib. | | | | | | |

Table S4.3 Results of Cox regression model with time to drug discontinuation as dependent variable and treatment group as independent variable. Including an interaction term between treatment group and ACPA status^a^. Drug discontinuation is defined by DAS28≥2.6 (no remission)^b^

|  | **Drug survival** | | | | |  |
| --- | --- | --- | --- | --- | --- | --- |
|  |  | **Unadjusted** |  |  | **Adjusted*** |  |
|  | ***HR*** | ***95% CI*** | ***P value*** | ***HR*** | ***95% CI*** | ***P value*** |
| TOFA mono #ACPA | *Reference group* |  |  |  |  |  |
| TOFA + csDMARD #ACPA | 0.94 | 0.54 1.66 | 0.84 | 0.84 | 0.48 1.45 | 0.52 |
| ADA mono #ACPA | 0.88 | 0.59 1.31 | 0.53 | 0.87 | 0.59 1.29 | 0.50 |
| ADA + csDMARD #ACPA | 0.74 | 0.49 1.12 | 0.15 | 0.76 | 0.51 1.14 | 0.18 |
| ^a^ACPA - is the reference  ^b^Drug discontinuation is defined by stop date of tofacitinib/adalimumab in combination with DAS≥2.6 (no remission)  *Adjusted for confounders: age at start TOFA/ADA, sex, smoking status, baseline DAS28, follow up time, previous b/tsDMARD use  ADA mono, adalimumab monotherapy; 95% CI, 95% confidence interval; csDMARD, conventional synthetic disease-modifying antirheumatic drug; DAS28, disease activity score assessed using 28 joints; HR, hazard ratio; TOFA, tofacitinib. | | | | | | |

Table S4.4 Results of Cox regression model with time to drug discontinuation^a^ as dependent variable and treatment group as independent variable. Including an interaction term between treatment group and ACPA status^b^

|  | **Drug survival** | | | | |  |
| --- | --- | --- | --- | --- | --- | --- |
|  |  | **Unadjusted** |  |  | **Adjusted*** |  |
|  | ***HR*** | ***95% CI*** | ***P value*** | ***HR*** | ***95% CI*** | ***P value*** |
| TOFA mono #ACPA | *Reference group* |  |  |  |  |  |
| TOFA + csDMARD #ACPA | 0.91 | 0.53 1.56 | 0.73 | 0.80 | 0.48 1.33 | 0.39 |
| ADA mono #ACPA | 0.95 | 0.65 1.39 | 0.80 | 0.93 | 0.65 1.33 | 0.69 |
| ADA + csDMARD #ACPA | 0.82 | 0.56 1.21 | 0.31 | 0.82 | 0.57 1.18 | 0.29 |
| ^a^Drug discontinuation is defined by stop date of tofacitinib/adalimumab  ^b^ACPA - is the reference  *Adjusted for confounders: age at start TOFA/ADA, sex, smoking status, baseline DAS28, follow up time, previous b/tsDMARD use  ADA mono, adalimumab monotherapy; 95% CI, 95% confidence interval; csDMARD, conventional synthetic disease-modifying antirheumatic drug; DAS28, disease activity score assessed using 28 joints; HR, hazard ratio; TOFA, tofacitinib. | | | | | | |

Table S4.5 Results of Cox regression model with time to drug discontinuation as dependent variable and treatment group as independent variable. Including an interaction term between treatment group and age^a^. Drug failure is defined by DAS28≥2.6 (no remission)^b^

|  | **Drug survival** | | | | |  |
| --- | --- | --- | --- | --- | --- | --- |
|  |  | **Unadjusted** |  |  | **Adjusted*** |  |
|  | ***HR*** | ***95% CI*** | ***P value*** | ***HR*** | ***95% CI*** | ***P value*** |
| TOFA mono #age | *Reference group* |  |  |  |  |  |
| **TOFA + csDMARD #age** | **1.01** | **0.99 1.03** | **0.09** | **1.02** | **0.99 1.04** | **0.07** |
| **ADA mono #age** | **1.02** | **1.004 1.03** | **0.01** | **1.02** | **1.001 1.03** | **0.04** |
| **ADA + csDMARD #age** | **1.02** | **1.01 1.03** | **0.01** | **1.02** | **1.004 1.03** | **0.01** |
| ^a^Age is continuous  ^b^Drug discontinuation is defined by stop date of tofacitinib/adalimumab in combination with DAS≥2.6 (no remission)  *Adjusted for confounders: sex, ACPA +, smoking status, baseline DAS28, follow up time, previous b/tsDMARD use  ADA mono, adalimumab monotherapy; 95% CI, 95% confidence interval; csDMARD, conventional synthetic disease-modifying antirheumatic drug; DAS28, disease activity score assessed using 28 joints; HR, hazard ratio; TOFA, tofacitinib. | | | | | | |

Table S4.6 Results of Cox regression model with time to drug discontinuation^a^ as dependent variable and treatment group as independent variable. Including an interaction term between treatment group and age^b^

|  | **Drug survival** | | | | |  |
| --- | --- | --- | --- | --- | --- | --- |
|  |  | **Unadjusted** |  |  | **Adjusted*** |  |
|  | ***HR*** | ***95% CI*** | ***P value*** | ***HR*** | ***95% CI*** | ***P value*** |
| **TOFA mono #age** | *Reference group* |  |  |  |  |  |
| TOFA + csDMARD #age | 1.01 | 0.99 1.03 | 0.11 | 1.01 | 0.99 1.03 | 0.13 |
| ADA mono #age | **1.01** | 0.99 1.02 | 0.13 | 1.01 | 0.99 1.02 | 0.14 |
| **ADA + csDMARD #age** | **1.01** | **0.99 1.02** | **0.09** | 1.01 | 0.99 1.02 | 0.11 |
| ^a^Drug discontinuation is defined by stop date of tofacitinib/adalimumab  ^b^Age is continuous  *Adjusted for confounders: sex, ACPA +, smoking status, baseline DAS28, follow up time, previous b/tsDMARD use  ADA mono, adalimumab monotherapy; 95% CI, 95% confidence interval; csDMARD, conventional synthetic disease-modifying antirheumatic drug; DAS28, disease activity score assessed using 28 joints; HR, hazard ratio; TOFA, tofacitinib. | | | | | | |

**SUPPLEMENTARY MATERIAL 5**

Information of multiple imputation model and the variables used:

ESR, CRP, PTGLBL, DAS28, DAS28_CRP, DAS28_ESR, Swollen Joint count,

baseline DAS28, age, RF, ACPA, smoking status (current/ever), sex, previous btsDMARD use (0, 1,≥2)
